# Identification and Characterization of a Novel Basic Helix-Loop-Helix Transcription Factor of Phospholipid Synthesis Regulation in *Aspergillus niger*

**DOI:** 10.3389/fmicb.2019.02985

**Published:** 2020-01-09

**Authors:** Hongzhi Dong, Dou Yu, Bin Wang, Li Pan

**Affiliations:** ^1^School of Biology and Biological Engineering, South China University of Technology, Guangzhou, China; ^2^Guangdong Provincial Key Laboratory of Fermentation and Enzyme Engineering, South China University of Technology, Guangzhou, China

**Keywords:** *Aspergillus niger*, *ino2*, transcription factor, EMSA, yeast two-hybrid assay, phosphatidylinositol, comparative transcriptome

## Abstract

The synthesis of phospholipids relies on a sort of genes, whose promoter regions contain inositol-sensitive upstream activation sequence (UAS_INO_) and are regulated by the basic helix-loop-helix (bHLH)-type *ino2/ino4* transcription factor (TF) pair. Ten putative bHLH TFs have been found through whole genome sequencing of *Aspergillus niger*, but none of these TFs have been characterized. In this study, we identified and characterized the bHLH-type TF *ino2(An02g04350)* in *A. niger.* Electrophoretic mobility shift assay (EMSA) and yeast two-hybrid assay demonstrated that *ino2* functions as a homodimer in UAS_INO_ genes (e.g., *ino1* and *cho1*) and binds to *opi1(An1g02370) in vitro*. Real-time quantitative PCR of *ino1* and quantification of total phospholipid indicated that the *ino2* disruptant downregulated the transcription of *ino1* and the amount of total cellular phosphatidylinositol. In addition, phenotype analyses showed that a loss of *ino2* led to resistance to cell wall interference and DNA damage. Comparative transcriptome analyses showed that more than 1000 genes and GO terms associated with UAS_INO_, endoplasmic reticulum–associated protein degradation, phosphatidylinositol synthesis, chitin synthesis, and fatty acid synthesis were differentially expressed in Δino2 compared to the wild type (WT). Taken together, these observations indicate that the bHLH TF *ino2* functions as a homodimer that regulates the synthesis of phosphatidylinositol, fatty acid, and chitin and influences the homeostasis of the endoplasmic reticulum membrane.

## Introduction

*Aspergillus niger* is a fungal cell factory marked by its great capacity for protein synthesis and secretion (Fleissner and Dersch, 2010). Its protein secretion pathways are constituted by the organelles of the endoplasmic reticulum, Golgi, vesicles, endosomes, and vacuole, which are composed of the phospholipid bilayer. Protein secretion among the endoplasmic reticulum, Golgi, and vesicles involves classic membrane trafficking, and phospholipid synthesis takes place mainly in the endoplasmic reticulum, Golgi, endosomes, and vacuole. Phospholipids can be divided into phosphatidylinositol (PI), phosphatidylserine (PS), phosphatidylcholine (PC), and phosphatidylethanolamine (PE; Wang et al., 2019). To date, phospholipid biosynthetic genes have been well characterized in *Saccharomyces cerevisiae.* However, their function in the filamentous fungi *A. niger* is largely unclear.

In the yeast model *S. cerevisiae*, the expression of phospholipid synthesis genes is controlled by the inositol-responsive *cis*-acting element [inositol-sensitive upstream activation sequence (UAS_INO_)] and the corresponding *trans*-acting factors (Ino2p, Ino4p, Opi1p; Carman and Han, 2011). Genes encoding enzymes in the CDP-DAG (e.g., *cds1*, *cho1*, *psd1*, *cho2*, *opi3*) and Kennedy (e.g., *eki1*, *ept1*, *cki1*, *cpt1*) pathways, genes involved in the synthesis of PI (e.g., *ino1*), as well as the genes encoding the inositol (*itr1*) and choline/ethanolamine (*hnm1*) permeases are coordinately regulated through a UAS_INO_ element in the promoter, which contains a basic helix-loop-helix (bHLH) TF binding site (Henry et al., 2012). The UAS_INO_ element binds the Ino2p-Ino4p heterodimer that activates transcription. Opi1p has an inhibitory effect on the Ino2p/Ino4p complex, and PA binds to Opi1p in the ER to deactivate its inhibition of Ino2p/Ino4p (Carman and Han, 2011). In wild-type strains and minimum culture medium, additional inositol leads to a rapid increase in PI synthesis, resulting in abundant consumption of PA and CDP-DAG. Opi1p is released from the ER and enters the nucleus, then binds to Ino2p, inhibiting Ino2p/Ino4p and deactivating ino1 and other UASino genes. When the concentration of inositol cannot satisfy the requirements for PI synthesis, a large amount of PA accumulates and binds Opi1p to the cytoplasm, consuming the opi1 in the nucleus and releasing the inhibition of Ino2p/Ino4p, thereby activating the expression of the UASino gene (Loewen et al., 2004). To date, 10 putative bHLH TFs have been found in *A. niger* through whole genome sequencing and analyses (Pel et al., 2007). However, the key transcription factors (TFs) regulating the expression of phospholipid synthesis genes are still unknown. Ino2p and Ino4p of yeast lack the corresponding homologous genes in the *A. niger* genome.

In this study, we identified and characterized a novel bHLH TF (*ino2*) in *A. niger*. Electrophoretic mobility shift assay (EMSA) and yeast two-hybrid assay indicated that, unlike in the Ino2p/Ino4p model in yeast, Ino2p functions as a homodimer to a canonical binding motif of the E-box (5′-CASSTG-3′) to regulate the UASINO genes (e.g., *ino1* and *cho1*). Comparative transcriptome and phenotype analyses of *ino2* disruptant indicated that *ino2* is also involved in endoplasmic reticulum–associated protein degradation, cell wall interference, and DNA damage response.

## Materials and Methods

### Strains, Media, and Conditions

*Escherichia coli Mach1T1* was used as the host strain for plasmid construction. *E. coli BL21(DE3)* was used as the host strain for recombination expression of proteins. The host strain *A. niger CBS513.88* (*kusA* deleted) was used for genetic manipulation. For *A. niger*, potato dextrose agar (PDA) solid medium was used for sporulation, high osmotic pressure Czapek–Dox (HCD) medium was used for protoplast transformation, and dextrose peptone yeast extract (DPY) liquid medium was used for DNA extraction. Strains, medium ingredients, and final concentrations of drugs used in this study are shown in Supplementary Tables S1 and S2.

### Construction of the *ino2* Gene-Disrupted Strain and ino2 Complementation Strain in *A. niger*

The *ino2* gene encodes a protein of 450 amino acids. We constructed the plasmids of gene knockout (p-ino2KO) and complementation (p-ino2C) using overlap extension PCR (OE-PCR), as shown in Supplementary Figure S1 p-ino2KO contained a 500 bp coding sequence (CDS) upstream, a 500 bp CDS downstream, a marker of *pyrG*, and a 1000 bp CDS downstream. p-ino2C contained a 500 bp CDS upstream, cDNA of ino2, the terminator of glucoamylase derived from *A. niger* (TglaA), a marker of *pyrG*, and a 1000 bp CDS downstream. The plasmid p-ino2KO was transformed the host strain CBS-1 and grown on HCD agar medium. Colonies of transformants were inoculated on CD agar medium after 4 days. Genomic DNAs obtained from the transformants were used as templates for amplification PCR. PCR results for primer sets F1/R1, F2/R2, F3/R3, and F4/R4 showed that the *ino2* gene was knocked out (Supplementary Figure S1B). We inoculated a correct transformant on PDA solid medium to collect the spores and named it “Δino2.” The plasmid p-ino2C was transformed into the strain Δino2 (obtained above) and grown on HCD agar medium. Colonies of transformants were inoculated on CD agar medium after 4 days. Genomic DNAs obtained from the transformants were used as the templates for amplification PCR. PCR results for primer sets F5/R5 and F6/R6 showed that the *ino2* gene was complemented successfully (Supplementary Figure S1B). We inoculated a correct transformant on PDA solid medium to collect the spores and named it “ino2C.” Plasmids used in this study are listed in Supplementary Table S3. Primers used in plasmid construction are listed in Supplementary Table S4. Polyethylene glycol/CaCl_2_-protoplast transformation was used for *A. niger* (Hinnen et al., 1978).

### Preparation of the 6xHis Fusion Protein and EMSA

We constructed expression plasmids of *An01g13950* and *An02g04350* in *E. coli* using pET22b (+; Addgene, Boston, MA, United States). The cDNA sequences of *An01g13950* and *An02g04350* were optimized and amplified with GenScript (Nanjing, China) and cloned into the pET22b (+) to generate pET22b-An01g13950 and pET22b-An02g04350. We induced expression of pET22b-An01g13950 and pET22b-An02g04350 in *E. coli* BL21 by adding 1 mM IPTG to a Luria–Bertani (LB) culture that had reached an OD_600_ of 0.6–0.8 at 37°C. After 16 h of incubation, the cells were harvested and lysed by sonication, and the recombination protein was purified by affinity chromatography using a Ni^+^ column. We eluted the recombination proteins using imidazole buffer and stored them at −80°C.

EMSAs were performed with biotin-labeled double-stranded oligonucleotides. The complementary single-stranded oligonucleotides listed in Supplementary Table S3 were synthesized by TIANYI HUIYUAN (Guangzhou, China) and annealed to generate double-stranded oligonucleotides. EMSA experiments were performed with a LightShift^TM^ Chemiluminescent EMSA Kit (Thermo Fisher Scientific, Waltham, MA, United States). Briefly, we used a 20 mL reaction volume that contained 2.5% glycerol (v/v), 5 mM MgCl_2_, 50 ng/μL poly(dI-dC), 0.05% NP-40 (v/v), 1X binding buffer, 20 fM biotin-labeled probe, and appropriate amounts of purification recombination protein. The reaction was performed at 25°C for 20 min. When required, unlabeled competitors were included immediately prior to the addition of the probe in the amounts indicated. Then the complexes were loaded on 6% polyacrylamide gel (29:1 crosslinking) with 0.5% TBE running buffer at 100 V. The following steps were performed according to the instructions in the LightShift^TM^ Chemiluminescent EMSA Kit.

### Yeast Two-Hybrid Assay

cDNA of nine bHLH genes (*An0204350*, *An03g04180*, *An03g05170*, *An08g01380*, *An08g04000*, *An09g06630*, *An14g02540*, *An15g03490*, and *An01g13950*) and *opi1(An15g02370)* were amplified. The PCR products were then constructed into the linearized plasmid pGADT7 AD (Takara, Otsu, Japan), which was digested with *Eco*RI and *Bam*HI using a NEBuilder^®^ HiFi DNA Assembly Master Mix (NEB, Ipswich, MA, United States). The nine plasmids were then transformed into the *S. cerevisiae* Y187 strain (Takara). The cDNA of *An01g13950* and *An02g04350* was amplified by PCR, and the PCR product was constructed into the linearized plasmid pGADT7 AD, which was digested with *Eco*RI and *Bam*HI. The plasmids pGBKT7-An01g13950 and pGBKT7-An02g04350 were then transformed the *S. cerevisiae* Y2HGold strain (Takara). The generated transformants were mixed and cultivated in 2 × YPDA liquid medium. The mated strains were cultured for 24 h and plated on selective solid medium DDO (SD/–Leu/–Trp dropout including every essential amino acid except for leucine and tryptophan) or QDO/x/A (SD/–Ade/–His/–Leu/–Trp dropout including every essential amino acid except for adenine, histidine, leucine, and tryptophan and supplemented with X-a-Gal and Aureobasidin A). Media used in Y2H are listed in Supplementary Table S2. Y2H experiment projects are listed in Supplementary Table S5.

### RNA Isolation, Quantitative Real-Time PCR, and RNA-Seq Analyses

For RNA isolation, *A. niger* strains were cultivated on CD liquid medium at 30°C and 200 rpm for 48 h. We collected mycelia using a suction flask and ground them in liquid nitrogen. We extracted total RNA using the HiPure Fungal RNA Kit (Magen, Guangzhou, China) according to the manufacturer’s instructions. We performed quantitative real-time PCR for *ino1(An10g00530)* using the designed primer sets listed in Supplementary Table S2, and glyceraldehyde-3-phosphate dehydrogenase *gpdA* was used as the reference gene. Gene expression was analyzed with 200 nM primer with the TransStart Tip Green qPCR SuperMix (Transgene, Beijing, China) and the 7500 Real-Time PCR Systems (Applied Biosystems, Foster City, CA, United States) according to the manufacturers’ instructions. After quantification and qualification, the total RNA was sequenced on the BGISEQ-500 platform.

Gene ontology (GO) term analysis was assigned with Cytoscape with the ClueGo plug-in as described previously (Zhou et al., 2016). GO terms with false discovery rate values <0.05 were defined as overrepresented. ClueGo analysis parameters were set as follows: View Style Setting as Significance, show only Pathways with *p* < 0.05, GO tree interval from Level 3 to Level 11. We calculated the significance of each term or group using a two-sided hypergeometric test and *p*-value correction using the Benjamini–Hochberg method. The corrected gene annotation list had 12,357 genes that were associated with one or more GO terms.

Homologs in *A. niger* were defined as follows. First, we found amino acid sequences in *S. cerevisiae* and then did a BLASTP search in the *A. niger* database to obtain the homologous scores. Homologs were selected as follows. If one of the genes scored much higher than the others, the highest scoring gene was chosen as the homolog; if there were many genes with similar high scores, the three highest scoring genes were selected. Supplementary Tables S9–S11 and S13–S15 list the homologs of UAS_INO_, fatty acid synthase, PI synthesis pathway, ER-associated protein degradation (ERAD), chitin synthesis, and DNA damage response genes in *A. niger.*

### Lipid Extraction and Phosphatidylinositol Detection Using LC-MS/MS

Spores were cultured in DPY liquid medium and the mycelia harvested after 48 h. Then 2 g mycelia and 10 mL liquid nitrogen were added to mortar and ground gently for 5 min. Then 7.5 mL chloroform: methanol (1:2) was added, ground for 2 min, and incubated for 2 min. The mortar was added to 2.5 mL chloroform and ground for 30 s, then added to 2.5 mL sterile water and ground for 30 s. The mixture was then transferred to a 50 mL tube and centrifuged at 7000 × *g* for 5 min. The lower organic phase was collected and transferred to a new 50 mL tube. Next 4 mL chloroform was added, and the mixture was shaken gently for a second extraction. The tube was centrifuged at 7000 × *g* for 5 min, and the lower organic phase was collected and transferred to a new 15 mL tube. The extract was dried with speed-Valco (Auto Science, Tianjin, China). The dried lipid extract was weighed, reconstituted in chloroform: methanol (1:1), and further diluted with HPLC mobile phase for HPLC/MS assay.

We performed quantification analyses of PI using a Shimadzu Nexera X2 LC 30AD ultra-high-performance liquid chromatography (UHPLC) system and a 4500 Q-Trap mass spectrometer (Applied Biosystems). We developed a sensitive method using a Kinetex^®^ 2.6 μm C18 100 Å LC column (100 × 2.1 mm). The mobile phase of UHPLC used the following binary gradients: (A) H_2_O: acetonitrile:0.5 M ammonium acetate (95:5:1) and (B) isopropanol: acetonitrile:0.5 M ammonium acetate (90:10:1). The gradient started at 0% B, increased linearly to 100% B over 1 min, held constant at 100% B for 6 min, returned to 0% B over 1 min, and held constant at 0% B for 4 min; the total run time was 11 min, and the flow rate was 0.5 mL/min. The column temperature was 25°C. The injection volume was 5 μL. Mass spectrometry was performed under negative ESI mode with multiple reaction monitoring (MRM) scan mode. ESI conditions were as follows: turbo source voltage: −4500 V; source temperature: 500°C; scan rate: 200 Da/s; GS1:50.0, GS2:50.0, curtain gas: 40.0; scan range: 100–1000 Da. Samples were resuspended in UHPLC phase A. To quantify PI in *A. niger*, we used a standard of L-α-phosphatidylinositol (Sigma; PI 34:2, 833.8/241.0) to optimize the parameters (declustering potential: −172.15 V, collision energy: −101.91). Counts per second (CPS) and peak area calculations were generated with Sciex Analyst 1.6.3 (Applied Biosystems).

## Results

### Identification of an ino2-Like bHLH Transcription Factor That Regulates Phospholipid Synthesis Genes in *Aspergillus niger*

**FIGURE 1:**
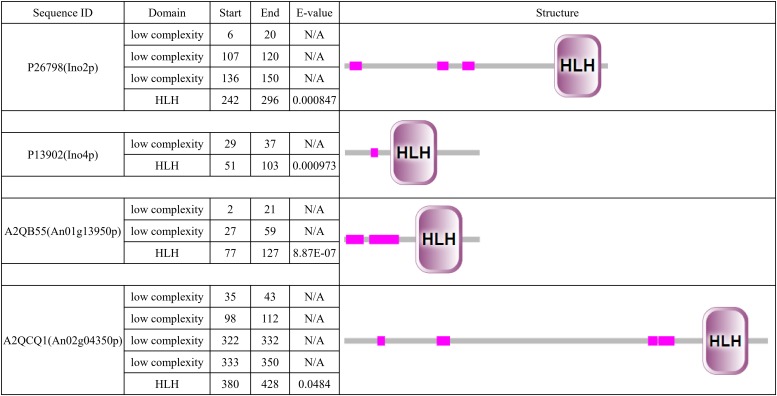
Simple modular architecture research tool (SMART) analysis of Ino2p, Ino4p, An01g13950p, and An02g04350p. Predicted HLH domain of the four proteins based on a SMART protein search (http://smart.embl-heidelberg.de/).

To determine the Ino2p/Ino4p-like bHLH TF in *A. niger*, we performed BLASTP analyses of the *A. niger* database with the *S. cerevisiae* Ino2p or Ino4p as a query. We identified nothing as the best hit of Ino2p and An01g13950p (e-value 2.0e-5, score 44) and An02g04350p (e-value 5.0e-4, score 39) as the best hits of Ino4p. It is interesting that these two proteins are contained in the 10 bHLH protein predicted previously in *A. niger* (Pel et al., 2007), which suggests that they are potential orthologs of Ino2p/Ino4p. Simple modular architecture research tool (SMART) analyses revealed that all four proteins contained low-complexity domains and a bHLH domain. In terms of protein length and domain distribution, we found that An02g04350p was closer to Ino2p (Figure 1).

**FIGURE 2:**
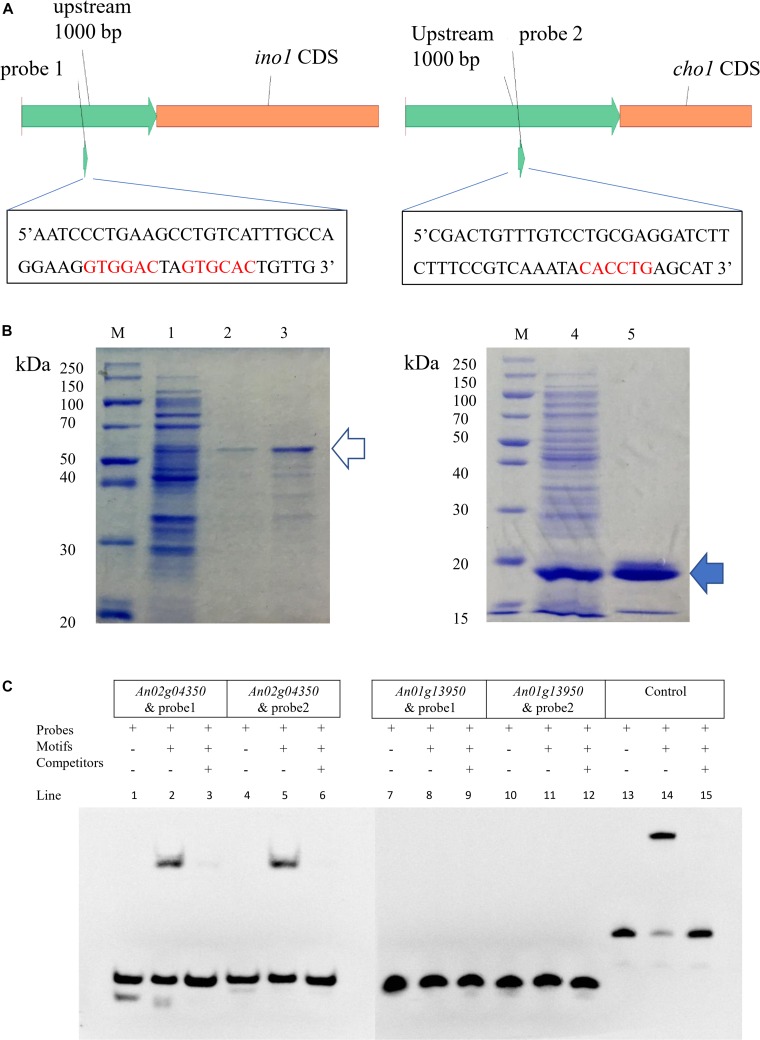
EMSA of *An01g13950* and *An02g04350* binding to the putative E-box. **(A)** Positions of putative E-boxes (5′-CASSTG-3′) in the 1000 bp upstream of the CDS of *ino1* and *cho2.* Sequences of the two probes are shown. **(B)** Expression of pET22b-An02g04350 and pET22b-An01g13950 in *E. coli* BL21(DE3). Line 1, intracellular protein of pET22b-An02g04350; line 2, elution of 500 mM imidazole of line 1, about 50 kDa, indicates the protein of An02g04350 (the hollow arrow); line 3, concentrate of line 2 by 30 kDa ultrafiltration; line 4, intracellular protein of pET22b-An01g13950; line 5, elution of 150 mM imidazole of line 4, about 15 kDa, indicates the protein of *An01g13950* (the solid arrow). **(C)** EMSA. Components are marked with + and –. Only lines 2 and 5 have shift bands, which indicate that the two probes can be combined with the protein of *An02g04350.* Lines 13–15 indicate the control EBNA system results: line 13, biotin-EBNA control DNA only; line 14, biotin-EBNA control DNA + EBNA extract; line 15, biotin-EBNA control DNA + EBNA extract +200-fold molar excess of unlabeled EBNA DNA. These combinations were specific because all shift bands disappeared after the addition of 200-fold unlabeled probes.

Research in *S. cerevisiae* has shown that *ino2/ino4* is the TF pair that regulates UAS_INO_ genes (Henry et al., 2012). A total of 22 predicted orthologous UAS_INO_ genes were present based on BLASTP analyses of the *A. niger* database with the *S. cerevisiae* UAS_INO_ genes as queries (Supplementary Table S9). *An10g00530* (homolog of *ino1*, responsible for the synthesis of inositol-3-phosphate) and *An01g09480* (homolog of *cho1*, responsible for the synthesis of phosphatidylserine) were chosen to search the motif of the E-box, and sequence analyses of the 1000 bp 5′ flanking region upstream of *ino1* and *cho1* revealed the presence of CASSTG for the E-box (GTGGAC, GTGCAC, and CACCTG). The two regions containing E-box were chosen as the probe (probe1 and probe2; Figure 2A). To confirm which of the two proteins (An01g13950p or An02g04350p) was the bHLH-like TF in *A. niger*, we performed EMSA to verify the binding of the two putative TFs and their motifs. cDNA of *An01g13950* and *An02g04350* was amplified and fused to the pET22b expression vector. Proteins were expressed in *E. coli BL21(DE3)* and purified using a 6xHis-tag (Figure 2B). The two target protein bands (marked with arrows in Figure 2) were analyzed by MALDI-TOF mass spectrometry, and the results indicated that the two bands were exactly the target TFs (Supplementary Table S8). The EMSA results in Figure 2C show that An02g04350p but not An01g13950p binds to either probe1 or probe2. Taken together, the results indicate that An02g04350p is the bHLH-like TF that regulates phospholipid synthesis genes in *A. niger* and that it may be the homolog of Ino2p in *S. cerevisiae.*

### Protein Interaction Analyses by Yeast Two-Hybrid Assay Indicate That *An02g04350* Is the Homolog of *ino2* and Functions as a Homodimer

**FIGURE 3:**
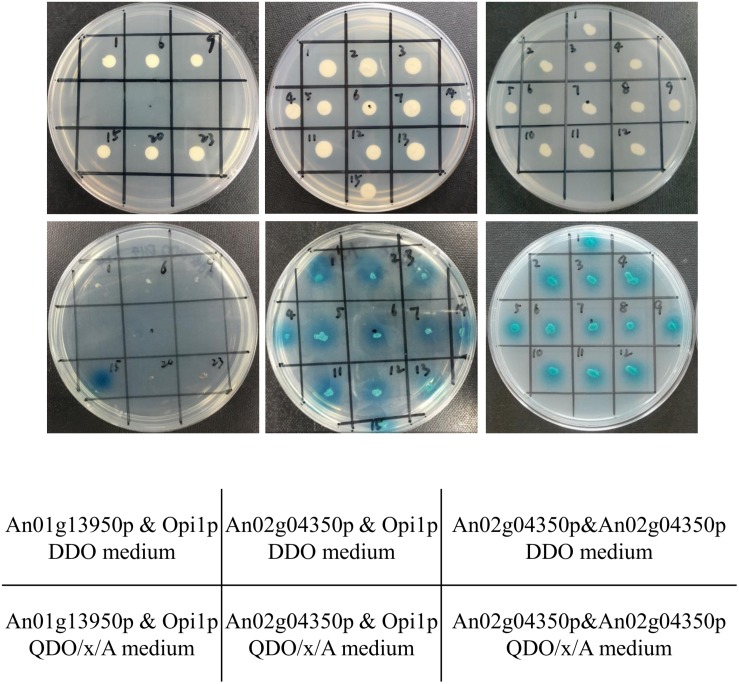
Confirmation of interactions of An01g13950p & Opi1p (An15g02370p), An02g04350p & Opi1, and An02g04350p & An02g04350p by yeast two-hybrid assay. The plasmids pGBKT7-An01g13950 and pGBKT7-An02g04350 were transformed into the *S. cerevisiae* Y2HGold strain, and pGADT7-opi1 and pGADT7-An02g04350 were transformed into the *S. cerevisiae* Y187 strain. Transformed yeast cells were grown on DDO selective solid medium and further inoculated on QDO/x/A selected solid medium.

Opi1p can combine Ino2p and repress genes regulated by *ino2/ino4*, such as *ino1*, the inositol-3-phosphate synthase (Loewen et al., 2004). In *S. cerevisiae*, regulation of *ino2/ino4* occurs at the transcription level and involves the Ino2p/Ino4p complex and the Opi1p repressor (Henry et al., 2012). Yeast two-hybrid assay was performed to further verify whether An01g13950p or An02g04350p binds Opi1p (An15g02370p) in *A. niger.* pGADT7-opi1 was constructed and transformed *S. cerevisiae* Y187, and pGBKT7-An01g13950 and pGBKT7-An02g04350 were constructed and transformed *S. cerevisiae* Y2HGold. The generated transformants were mixed, and the mated strains were cultured in YPDA for 24 h and then plated on DDO solid medium. Positive colonies were further inoculated on QDO/X/A solid medium. Figure 3 shows that An02g04350p but not An01g13950p combines with *opi1.* This proves that *An02g04350* is the homolog of *ino2* but not *ino4* and that *An02g04350* should be named “*ino2*.”

In *Aspergillus*, bHLH proteins usually form homodimers (Caruso et al., 2002) or heterodimers (Jin et al., 2011b) with other bHLH proteins while binding to the E-box and regulating target genes (Jin et al., 2011a). To confirm the other heterodimer of *ino2*, we performed yeast two-hybrid assay with pGBKT7-AN02g04350, pGADT7-An02g04350, and the eight remaining bHLH TFs (pGADT7-An01g13950, pGADT7-An03g05170, pGADT7-An08g01380, pGADT7-An08g04000, pGADT7-An09g06630, pGADT7-An14g02540, pGADT7-An15g03490, and pGADT7-An01g13950). PGBKT7-An02g04350 transformed the *S. cerevisiae* Y2HGold strain, and the nine pGADT7 plasmids transformed *S. cerevisiae* Y187. The generated transformants were mixed as described in Supplementary Table S5, and the mated strains were cultured for 24 h and plated on DDO solid medium. Positive colonies were further inoculated on QDO/X/A solid medium. Results indicated that none of these eight bHLH proteins but pGADT7-An02g04350 combined with pGBKT7-An02g04350 (Figure 3 and Supplementary Figure S2). Combined with the EMSA results shown in Figure 2C, these findings indicate that An02g04350p binds E-box motifs of *ino1* and *cho1* by itself, which further indicates that bHLH TF *ino2* functions as a homodimer *in vitro.*

### Ino2 Disruptant Suppresses the Transcription of Inositol-3-P Synthase *ino1* and Influences the Production of PI in *A. niger*

**FIGURE 4:**
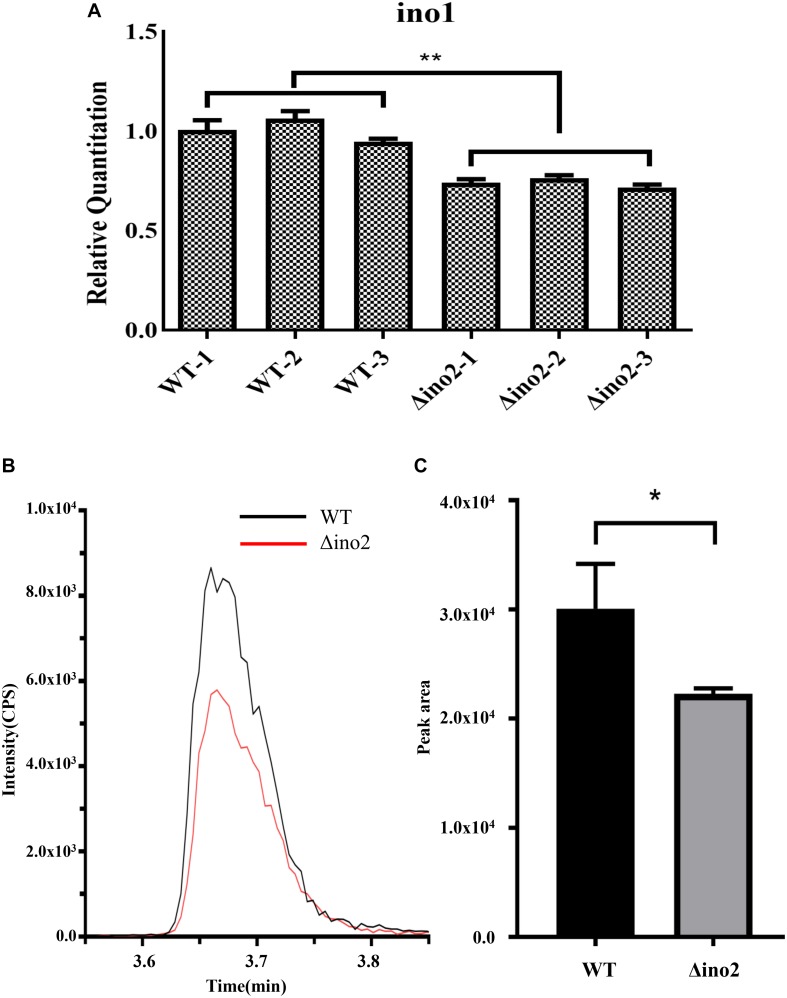
Comparative qRT-PCR of *ino1* and PI concentration analyses of WT and Δino2. **(A)** The relative expression of *ino1* was examined and compared to qRT-PCR. Expression was normalized to the expression of the endogenous control gene *gpdA.* The expression of *ino1* in WT was used as the baseline. Each experiment was performed with three biological replicates (e.g., WT-1, WT-2, and WT-3) and four technical replicates. ^∗∗^*p* < 0.005 compared to WT. *p*-values were determined with Student’s *t*-test. **(B,C)** Comparative analyses of PI concentration between WT and Δino4 by UHPLC-MS/MS in the MRM mode. Ion intensity and the peak area of PI 34:2 (833.4/241) were monitored with Sciex Analyst 1.6.3. Each experiment was performed with three biological replicates. ^∗^*p* < 0.05 compared to WT. *p*-values were determined with Student’s *t*-test.

To further explore the regulation mechanism of *ino2* in *A. niger*, we constructed the *ino2* disruptant (Δino2) and *ino2* complementation (ino2C) as shown in Supplementary Figure S1. In *S. cerevisiae*, the ino2p/ino4p complex binds to the promoter of *ino1* (inositol-3-P synthase) and positively regulates the biosynthesis of phospholipid (Ambroziak and Henry, 1994). To validate the regulation relationship of *ino2* and *ino1* in *A. niger*, we investigated the transcription of inositol-3-phosphate synthase gene *ino1* by comparative quantitative reverse transcriptase PCR (qRT-PCR) between Δino2 and the wild type (WT). Spores of Δino2 and the WT strain were cultivated on CD liquid medium at 30°C and 200 rpm for 48 h, and mycelia were collected to extract the total RNA as described in Materials and Methods. Primer set F-gpdA/R-gpdA was used as an internal standard, and F-ino1/R-ino1 was used to detect the transcription of *ino1.* Results showed that compared to WT, transcription of *ino1* was repressed in Δino2 (Figure 4A).

Phosphatidylinositol is critical in the formation of the fungal membrane. In *S. cerevisiae*, the inositol used in PI synthesis is either synthesized *de novo* or obtained from the growth medium (Nikawa et al., 1991). *Ino1* is an indispensable gene in the biosynthetic pathway of PI. To detect change in PI in Δino2 compared to WT, we extracted total phospholipid from logarithmic mycelium and quantified it using high-performance liquid chromatography coupled with mass spectrometry (LC-MS) as described in Materials and Methods. The intensity ratio of PI 34:2 showed that PI was lower in Δino2 than in WT. Quantification analyses of the peak area showed about a 25% decrease in Δino2 compared to WT (Figures 4B,C, Supplementary Figure S3, and Supplementary Table S6).

### Disruption of *ino2* Shows Phenotypic Resistance to Cell Wall Interference and DNA Damage Compared to the WT Strain

**FIGURE 5:**
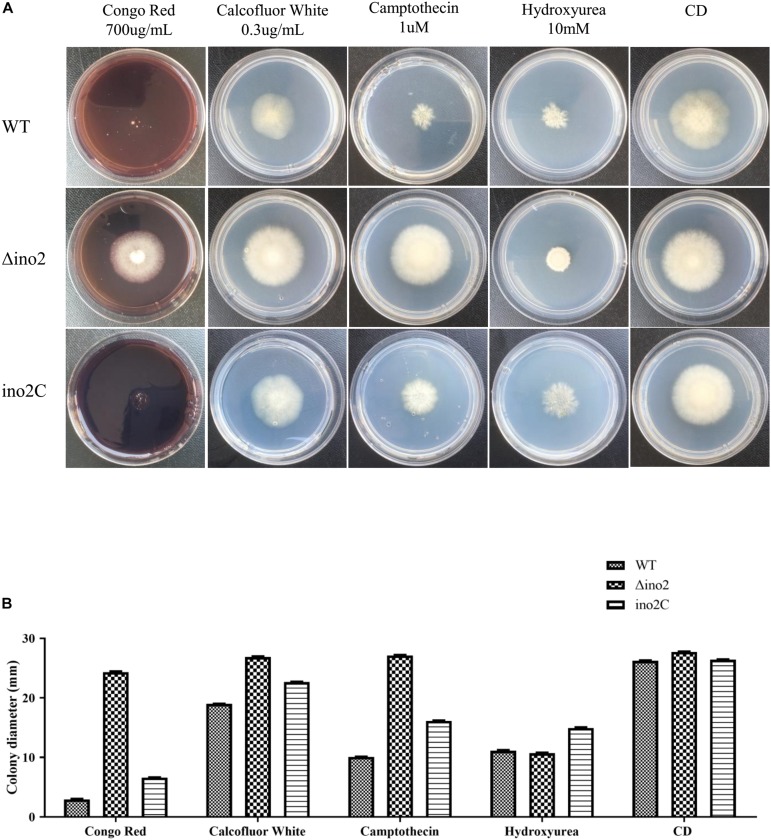
Comparisons of the phenotypes of the WT, ino2 disruptant, and ino2 complementation strains. **(A)** The WT, Δino2, and ino2C strains were inoculated on five CD solid media (CD with 700 μg/mL Congo red, CD with 0.3 μg/mL calcofluor white, CD with 1 μ camptothecin, CD with 10 mM hydroxycarbamide, and CD only) at 30°C for 5 days. **(B)** The diameters of colonies in panel **(A)**.

To evaluate the influence of *ino2* on the phenotype of *A. niger*, we cultured an equivalent concentration of spores of Δino2 strain on CD solid medium with four types of drugs (Congo red and calcofluor white for cell wall interference, and camptothecin and hydroxyurea for DNA damage; Lima et al., 2005; Malavazi et al., 2006; Ram and Klis, 2006) and compared them to ino2C and WT. After 7 days of cultivation, diameters of colonies were measured. Results showed that the colonies of Δino2 were larger than those of WT and ino2C on CD with Congo red, calcofluor white, and camptothecin. The colonies on CD with hydroxyurea were more compact than those of WT and ino2C strains. These resistances were specific because all phenotypes were restored in ino2C compared to WT (Figures 5A,B).

### Comparative Transcriptome Analysis of Δino2, INO, and WT

To identify genes differentially expressed in Δino2 compared to WT, we extracted total RNA from the logarithmic mycelium of different strains with different cultures. Spores of the Δino2 strain were inoculated into liquid CD medium, and spores of the WT strain were inoculated into liquid CD or CD + 100 mg/L inositol to extract total RNA and prepare the RNA-seq as described in section “Materials and Methods.” The three samples were named “Δino2” (Δino2 strain with liquid CD medium), “INO” (WT strain with liquid CD + 100 mg/L inositol), and “WT” (WT strain with liquid CD medium). Detailed descriptions are listed in Supplementary Table S7. We normalized gene expression levels as FPKM (fragments per kilobase million) mapped reads to compare the gene expression of each sample. Differentially expressed genes (DEGs) were defined as statistically significant when the false discovery rate <0.05. The DEG analysis between Δino2 and WT identified 2099 DEGs. Of these, 1183 genes were induced and 916 genes were repressed in Δino2. The GO function classification of DEGs indicated that the glucosamine-containing compound catabolic process, choline dehydrogenase activity, the lipid catabolic process, and fatty acid synthase activity were induced, whereas genes associated with ion and ammonium transmembrane transport activity and the cell wall were repressed (Supplementary Figure S4). Results indicated that compared to WT, Δino2 affects the steady state of the membrane structure in *A. niger.* The DEG analysis between INO and WT identified 773 DEGs. Of these, 441 genes were induced and 332 genes were repressed in INO.

In *S. cerevisiae*, the Ino2p/Ino4p complex regulates UAS_INO_ genes and fatty acid–associated genes (UAS_FAS_; Schüller et al., 1992). To evaluate the importance of *ino2* with respect to UAS_INO_ genes in *A. niger*, we found 22 orthologs of UAS_INO_ genes in the ASPGD database. Compared to WT, 16 UAS_INO_ genes were repressed in Δino2, which is similar to the results for *S. cerevisiae* (Supplementary Table S9) (Greenberg and Lopes, 1996). Compared to WT, 17 UAS_INO_ genes were repressed in INO, which is similar to the results for Δino2. Moreover, 12 homologous genes of fatty acid synthesis were found in the ASPGD database and listed. However, compared to WT, 11 genes were upregulated in Δino2 (Supplementary Table S10).

In *S. cerevisiae*, the PI *de novo* synthesis pathway comprises three enzymes: inositol-3-phosphate synthase *ino1*, inositol monophosphate *inm1*, and phosphatidylinositol synthase *pis1* (Henry et al., 2012). Homologs were found in *A. niger* and compared between Δino2 and WT to assess the transcription of these three genes. Results showed that *ino1* was downregulated, *inm1(An03g03700)* was upregulated, and *pis1(An01g14140)* was almost stable (Supplementary Table S11). GO enrichment analyses were performed to gain insight into the process that affects PI synthesis in Δino2. Results showed that genes in GO terms positive to PI synthesis (GO:0006661 and GO:0010513) were mostly (80%) downregulated, and genes negative to PI synthesis were upregulated (Supplementary Table S12).

To evaluate the influence of the *ino2* gene on the homeostasis of ER, we found 16 genes homologous to *S. cerevisiae* correlated with ERAD in *A. niger* and compared their expression between Δino2 and WT (Supplementary Table S13). Results showed that 10 out of the 16 genes were repressed.

In the GO function classification of DEGs, several GO groups associated with the synthesis of the fungal cell wall varied significantly: 33% of genes associated with the glucosamine-containing compound catabolic process were induced, and 13.2% of genes associated with the fungal-type cell wall and 33% of cell wall–bounded periplasmic space genes were repressed (Supplementary Figure S4). Previous phenotype analyses of Congo red and calcofluor white showed that Δino2 is more resistant to cell wall interference than WT. Homologous genes associated with chitin synthesis were found in *A. niger* to assess the importance of *ino2* to genes associated with chitin metabolism and the fungal-type cell wall. Comparative transcriptome analyses of these genes showed that genes in GO:0034221 and homologs of *chs3*, *chs5*, and *chs7* were mostly repressed in Δino2 and INO compared to WT (Supplementary Table S14).

## Discussion

### Identification of the Novel bHLH Type TF *ino2* in *Aspergillus niger*

Basic helix-loop-helix protein is a family that binds to a consensus core sequence called the E-box (5′-CASSTG-3′), and the G-box (5′-CACGTG-3′) is the most common form (Wang et al., 2015). We report here a newly identified bHLH family TF, *ino2(An02g04350)*, in *A. niger.* EMSA demonstrated that *ino2* binds with E-box motif (CASSTG), which is similar to other bHLH TFs in *Aspergillus* (ecdR, devR, sclR, snBH1, and stuA; Dutton et al., 1997; Caruso et al., 2002; Jin et al., 2011a, b; Zhuang et al., 2019). Yeast two-hybrid assay demonstrated that *ino2* does not bind *in vitro* with the eight other bHLH TFs previously discovered (*An08g04000*, *An15g03490*, *An01g13950*, *An09g06630*, *An03g04180*, *An08g01380*, *An14g02540*, and *An03g05170*; Pel et al., 2007) by itself in *A. niger.* In *S. cerevisiae*, *ino2* and *ino4* are two bHLH family TFs, the proteins of which form the heterodimer Ino2p/Ino4p complex to activate genes of phospholipid biosynthesis containing UAS_INO_ (Graves and Henry, 2000). *Opi1* (over producer of inositol) is a transcriptional regulator of the Ino2p/Ino4p complex that binds with the repressor interaction domain of *ino2* and represses activation of UAS_INO_ (Villa-García et al., 2011). Here, we used yeast two-hybrid assay to verify the *in vitro* combination of *ino2* and *opi1(An15g02370)* in *A. niger*, which is similar to findings for *S. cerevisiae.*

In *S. cerevisiae*, Ino2p and Ino4p form a heterodimer to bind the E-box and activate UAS_INO_ genes (Graves and Henry, 2000). After searching the ASPGD database, we found two putative orthologs of *ino4* (*An01g13950* and *An02g04350*) in *A. niger* but no ortholog of *ino2.* EMSA results for the two putative *ino4* and two probes (E-box in *ino1* and *cho1*) showed that An02g04350p but not An01g13950p binds with the E-boxes. Furthermore, results of yeast two-hybrid assay demonstrated that An02g04350p binds with Opi1p and itself separately *in vitro*, which further suggests that *An02g04350* is the ortholog of *ino2* and functions as a homodimer, which is similar to findings for *Aspergillus nidulans AnBH1* (Caruso et al., 2002).

### Characterization of the Novel TF *ino2* by Phenotype and Comparative Transcriptome Analyses

Genes containing UAS_INO_, called UAS_INO_ genes, are important in regulating phospholipid synthase as well as genes associated with fatty acid synthesis (*fas1*, *fas2*, and *acc1*) in *S. cerevisiae* (Jesch et al., 2005). Previous comparative transcriptome analyses indicated that the transcription of these genes was positively related to *ino2* and/or *ino4* (Chumnanpuen et al., 2013). In the present study, 22 predicted homologs of UAS_INO_ genes were found in *A. niger.* Among them were three genes (cpt1, eki1, and ept1) that shared three homologs (An01g11150, An11g09660, and An16g07870) because of incomplete annotation in *A. niger.* Compared to WT, 16 of 22 UAS_INO_ genes were downregulated in Δino2, which indicates that the disruptant of *ino2* reduces transcription of UAS_INO_ genes. It is interesting that compared to WT, 11 of 16 UAS_INO_ genes in INO showed reduced transcription, which indicates that additional inositol represses the expression of UAS_INO_ genes, which is similar to results for *S. cerevisiae* (Ashburner and Lopes, 2015). *Fas1*, *fas2*, and *acc1* are necessary for the *de novo* synthesis of long-chain saturated fatty acids. Fourteen homologs of *fas1* and *fas2* were found in *A. niger*, and transcription was compared between Δino2 and WT. Results indicated that 12 out of these 14 genes were upregulated, which contradicts previous results for *S. cerevisiae* that the *ino2* gene positively regulates *fas1* and *fas2* (Schüller et al., 1992).

The synthesis of PI, an important component in membrane formation in fungal cells, is regulated in response to the phospholipid precursor inositol. Inositol-3-P synthase *ino1* is irreplaceable in the *in vivo* synthetic pathway of inositol (Dean-Johnson and Henrye, 1989). In *S. cerevisiae*, high *ino2/ino4* transcription increases the fluxes through the CDP-DAG and phospholipid synthesis pathways, which support the synthesis of PI, and also enhances the Kennedy pathway to produce more PC using choline and DAG as precursors (Chumnanpuen et al., 2013). In this study, a comparison of the transcription level *ino1* and total cellular PI between Δino2 and WT indicated that an absence of *ino2* decreased transcription of *ino1*, which consequently reduced the amount of total cellular PI. Transcriptome analyses of Δino2 showed that genes in GO terms positive to PI synthesis (GO:0006661 and GO:0010513) were mostly (80%) downregulated, and genes negative to PI synthesis were upregulated compared to WT. This result confirms that the disruptant of *ino2* represses the synthesis of PI, thus interrupting the formation of the fungal-type membrane. It is interesting that the addition of extra inositol decreased the transcription of *ino1* and *pis1* (phosphatidylinositol synthase) in the *ino2* disruptant strain (Supplementary Figure S5), which indicates that adding inositol represses expression of *ino1* independently of the *ino2* TF to some extent.

In *S. cerevisiae*, deletion of *ino2/ino4* results in amino acid starvation and then downregulates UPR and ERAD genes (for less missed fold proteins). Moreover, 16 homologs associated with ERAD genes were found in Jonikas’s research (Jonikas et al., 2009). A transcriptional comparison between Δino2 & WT and INO & WT showed that 10 and 12 genes were downregulated, which demonstrates that the disruption of *ino2* and addition of inositol might repress the ERAD response, similar to findings reported for *S. cerevisiae* (Jesch et al., 2005). Decreasing phospholipid levels leads to the accumulation of saturated phospholipids in the ER membrane, which activates UPR and leads to upregulation of fatty acid, triacylglycerol, and sterol biosynthesis. This indicates that the bHLH TF *ino2* plays an important role in regulating synthesis of fatty acids and phospholipids and thus influences UPR and ERAD in the ER membrane in *A. niger.*

The fungal-type cell wall is composed of β-1,3-glucan, chitin, and cell wall proteins (Fontaine et al., 2000). The presence of Congo red and calcofluor white result in swelling or lysis of hyphal tips in *A. niger*, which in turn activates the cell wall stress response to resist drugs (Ram and Klis, 2006). Phenotype analyses of Δino2 compared to ino2 complementary (ino2C) and WT indicate that Δino2 shows great resistance to Congo red and calcofluor white. Results of comparative transcriptome analyses show that the homologous genes associated with chitin synthase (*chs3*, *chs5*, and *chs7*) are downregulated. These results are consistent with the phenomenon in *S. cerevisiae.*

In fungal-type cells, the presence of camptothecin or hydroxyurea generates DNA damage (Malavazi et al., 2006; Son et al., 2016). Camptothecin generates replication-mediated double-strand breaks, which induce cell cycle arrest in G_2_–M. To assess the phenotype of Δino2 in camptothecin or hydroxyurea, we measured the transcription of six homologs associated with DNA damage repair (*mshA*, *cshA*, *fhdA*, *tprA*, *uvsC*, and *top1*) in *A. niger* (Bruschi et al., 2001; Malavazi et al., 2006). Results showed that these six genes were repressed in Δino2 compared to WT (Supplementary Table S15), which contradicts the findings in *A. nidulans.* We speculate that the reason for this is that the disruptant of *ino2* causes an imbalance in membrane homeostasis, which makes the nuclear membrane inaccessible, thus preventing damage to genomic DNA by these drugs.

## Data Availability Statement

The datasets generated for this study can be found in the Sequence Read Archive submission: SUB5955242.

## Author Contributions

BW and LP conceived and designed the work. HD and DY performed the experiments. LP and HD analyzed the data. HD wrote the manuscript. LP and BW revised the manuscript for intellectual content. All authors have read and agreed to the submission of the manuscript.

## Conflict of Interest

The authors declare that the research was conducted in the absence of any commercial or financial relationships that could be construed as a potential conflict of interest.
