# Supplementary material for: Identification and Characterization of a Novel Basic Helix-Loop-Helix Transcription Factor of Phospholipid Synthesis Regulation in Aspergillus niger
Source: Front Microbiol. 2020 Jan 9;10:2985. doi: 10.3389/fmicb.2019.02985 (PMC6962311; doi:10.3389/fmicb.2019.02985)
Supplement: Supplementary file 1 [file Data_Sheet_1.docx]

1. **Supplementary tables.**

**Table S1. Strains used in this study**

| **Strains** | **Genotype and Source** |
| --- | --- |
| WT | *Aspergillus niger CBS 513.88* |
| CBS-1 | *ΔkusA,* derivate from WT |
| Δino2 | *ΔkusA; Δino2;* derivate from CBS-1 |
| ino2C | ino2 complementation, derivate from Δino2 |

**Table S2.** **Ingredient of cultures used in this study**

| **Culture** | **Ingredient** |
| --- | --- |
| PDA | 40 g of PDA powder; 5 g of agar per litre |
| DPY | 5 g of yeast extract, 10 g of peptone, 20 g of glucose, 5 g of KH_2_PO_4_, and 0.5 g of MgSO_4_·7H_2_O per litre |
| CD | 10 g of glucose, 3 g of NaNO_3_, 2 g of KCl, 0.5 g of MgSO_4_·7H_2_O, 1 g of K_2_HPO_4_·3H_2_O, 0.1 g of FeSO_4_·7H_2_O, and 20 g of agar per litre; pH 5.5 |
| HCD | 342.3 g of sucrose, 3 g of NaNO_3_, 2 g of KCl, 0.5 g of MgSO_4_·7H_2_O, 1 g of K_2_HPO_4_·3H_2_O, 0.1 g of FeSO_4_·7H_2_O, and 20 g of agar per litre; pH 5.5 |
| LB | 10 g of tryptone, 5 g of yeast extract, and 10 g of sodium chloride per litre |
| YPD | 10 g of yeast extract, 20 g of peptone, 20 g of glucose per litre |
| DDO | SD/–Leu/–Trp dropout including every essential amino acid except for leucine and tryptophan, which is used to select for the bait and prey plasmids. Devirated from Matchmaker® Gold Yeast Two-Hybrid System |
| QDO/x/A | SD/–Ade/–His/–Leu/–Trp dropout including every essential amino acid except for adenine, histidine, leucine, and tryptophan, and for the activation of the Gal-responsive HIS3 and ADE2 genes as part of the confrmation step of the two-hybrid, supplemented with X-a-Gal and Aureobasidin A. Devirated from Matchmaker® Gold Yeast Two-Hybrid System |

**Table S3.** Plasmids **used in this study**

| **Plasmids** | **Description/derivation** | **Reference** |
| --- | --- | --- |
| pMD20 | Routine cloning vector | Takara |
| p-ino2KO | *ino2* knockout frame inserted into pMD20 | This work |
| p-ino2C | *ino2* complementation frame into pMD20 | This work |
| pET22b | Routine cloning vector for protein expression in *Escherichia coli* | Takara |
| pET22b-An01g13950 | *An01g13950* cDNA inserted into pET22b | This work |
| pET22b-An02g04350 | *ino2* cDNA inserted into pET22b | This work |
| pGADT7 | Routine cloning vector for Yeast two-hybrid analysis | Takara |
| pGADT7-An02g04350 | *An02g04350* cDNA inserted into pGADT7 | This work |
| pGADT7-An03g04180 | *An03g04180* cDNA inserted into pGADT7 | This work |
| pGADT7-An03g05170 | *An03g05170* cDNA inserted into pGADT7 | This work |
| pGADT7-An08g01380 | *An08g01380* cDNA inserted into pGADT7 | This work |
| pGADT7-An08g04000 | *An08g04000* cDNA inserted into pGADT7 | This work |
| pGADT7-An09g06630 | *An09g06630* cDNA inserted into pGADT7 | This work |
| pGADT7-An14g02540 | *An14g02540* cDNA inserted into pGADT7 | This work |
| pGADT7-An15g03490 | *An15g03940* cDNA inserted into pGADT7 | This work |
| pGADT7-An01g13950 | *An01g13950* cDNA inserted into pGADT7 | This work |
| pGADT7-opi1 | *Opi1* cDNA inserted into pGADT7 | This work |
| pGBKT7 | Routine cloning vector for Yeast two-hybrid analysis | Takara |
| pGBKT7-An01g13950 | *An01g13950* cDNA inserted into pGBBKT7 | This work |
| pGBKT7-An02g04350 | *An02g04350* cDNA inserted into pGBBKT7 | This work |

**Table S4.** **Oligonucleotide primers used in this study**

| **Primer** | **Sequences** | **Experiment** |
| --- | --- | --- |
| F1 | CCAGCCTACGAATAAGATGC | Upstream location of p-ino2KO in the *ino2* locus |
| R1 | ACCCGACTATCATTCAAACAG | Upstream location of p-ino2KO in the *ino2* locus |
| F2 | ATGGTTCGCTGTACTTGCTT | Downstream location of p-ino2KO in the *ino2* locus |
| R2 | GGGTAAACTCGGTCTGCTT | Downstream location of p-ino2KO in the *ino2* locus |
| F3 | TGGCTGTAAGTGATTTCTTTAGTTG | Detection of *ino2* CDS |
| R3 | TGAGCTGTTGGGCTGTTTC | Detection of *ino2* CDS |
| F4 | CGCCTGAACAACAGCAAAC | Detection of *ino2* CDS |
| R4 | GCACAGATCATCGAACCCT | Detection of *ino2* CDS |
| F5 | TCCCGTCTATCTGTCAGTTTCA | Upstream location of p-ino2C in the *ino2* locus |
| R5 | GGTGTTCTGCTGGTAGTGGTC | Upstream location of p-ino2C in the *ino2* locus |
| F6 | CAGGTACTGCTGGATGAGGA | Downstream location of p-ino2C in the *ino2* locus |
| R6 | TCAATGAAGAACGGGAAGAT | Downstream location of p-ino2C in the *ino2* locus |
| F-probe1 | AACGGTATTGACTAAAAGGGAATCCCTGAAGCCTGTCATTTGCCAGGAAGGTGGACTAG | Amplification of probe1 |
| R-probe1 | CCAGGAAGGTGGACTAGTGCACTGTTGTTAATTAATATAAATACTGGCAAGGGATGCC | Amplification of probe1 |
| F-probe2 | AACGGTATTGACTAAAAGGGCGACTGTTTGTCCTGCGAGGATCTTCTTTCCGTCAAATA | Amplification of probe2 |
| F-probe2 | CTTCTTTCCGTCAAATACACCTGAGCATTTAATTAATATAAATACTGGCAAGGGATGCC | Amplification of probe2 |
| F-gpdA | TCTGCTCCTTCCGCTGATGC | Internal standard of RT-qPCR between WT and Δino2 |
| R-gpdA | ACGATGCCGAACTTGTCGTT | Internal standard of RT-qPCR between WT and Δino2 |
| F-ino1 | TGCTGGGCTCATGTTGAGAAGA | Detection of RT-qPCR between WT and Δino2 |
| R-ino1 | CTTGATAGCGTTGAGGAGGTTG | Internal standard of RT-qPCR between WT and Δino2 |
| F-pET22b-An01g13950 | GTGGTGGTGGTGGTGGTTGCGAAACGAATCATCAATGCGC | Construction of plasmid pET22b-An01g13950 |
| R-pET22b-An01g13950 | GAAGGAGATATACATATGCCATCTGCACGACCTCCTGC | Construction of plasmid pET22b-An01g13950 |
| F-pET22b-An02g04350 | GTGGTGGTGGTGGTGCCGGGGCATCACCAAGCCATTC | Construction of plasmid pET22b-An02g04350 |
| R-pET22b-An02g04350 | GAAGGAGATATACATATGGAACTAAACCGGTCGGGACAA | Construction of plasmid pET22b-An02g04350 |
| F-pGADT7-An02g04350 | GTACCAGATTACGCTGAACTAAACCGGTCGGGACAACAT | Construction of plasmid pGADT7-An02g04350 |
| R-pGADT7-An02g04350 | GTTTTTCAGTATCTACCGGGGCATCACCAAGCCATTC | Construction of plasmid pGADT7-An02g04350 |
| F-pGADT7-An03g04180 | GTACCAGATTACGCTATGTCCGATCCGTCGTTGGGTGC | Construction of plasmid pGADT7-An03g04180 |
| R-pGADT7-An03g04180 | GTTTTTCAGTATCTATTAAACCTGGCCTTGGTCAATGTCA | Construction of plasmid pGADT7-An03g04180 |
| F-pGADT7-An03g05170 | GTACCAGATTACGCTATGGCACTGCCCACCATGGGGA | Construction of plasmid pGADT7-An03g05170 |
| R-pGADT7-An03g05170 | GTTTTTCAGTATCTATCACGACCTTCTCGGGTACTTTTTC | Construction of plasmid pGADT7-An03g05170 |
| F-pGADT7-An08g01380 | GTACCAGATTACGCTATGATGTCACAGTCGAATTATATGTCC | Construction of plasmid pGADT7-An08g01380 |
| R-pGADT7-An08g01380 | GTTTTTCAGTATCTACTAATTCATCTCCATCGCATACTCG | Construction of plasmid pGADT7-An08g01380 |
| F-pGADT7-An08g04000 | GTACCAGATTACGCTATGTCACGCTCTCGTCTGCCACC | Construction of plasmid pGADT7-An08g04000 |
| R-pGADT7-An08g04000 | GTTTTTCAGTATCTATTAGGAAATAAGCAAGTCTTTAACGCT | Construction of plasmid pGADT7-An08g04000 |
| F-pGADT7-An09g06630 | GTACCAGATTACGCTATGGAGCAAGAGCACCCTCTGGC | Construction of plasmid pGADT7-An09g06630 |
| R-pGADT7-An09g06630 | GTTTTTCAGTATCTACTATGCAGAGTTGTCTATTTCATGAC | Construction of plasmid pGADT7-An09g06630 |
| F-pGADT7-An14g02540 | GTACCAGATTACGCTATGGCATACCCCAGGCCCGATTC | Construction of plasmid pGADT7-An14g02540 |
| R-pGADT7-An14g02540 | GTTTTTCAGTATCTATTAAGCGTGAAACGCCTCACTCCC | Construction of plasmid pGADT7-An14g02540 |
| F-pGADT7-An15g03490 | GTACCAGATTACGCTATGGAGACCACTCTTGCTCATCGA | Construction of plasmid pGADT7-An15g03490 |
| R-pGADT7-An15g03490 | GTTTTTCAGTATCTATCATTTATCATCAATAATAGGGAAAGAAA | Construction of plasmid pGADT7-An15g03490 |
| F-pGADT7-An01g13950 | GTACCAGATTACGCTCCATCTGCACGACCTCCTGCCT | Construction of plasmid pGADT7-An01g13950 |
| R-pGADT7-An01g13950 | GTTTTTCAGTATCTAGTTGCGAAACGAATCATCAATGCGC | Construction of plasmid pGADT7-An01g13950 |
| F-pGADT7-opi1 | GTACCAGATTACGCTGACTACCGCTCCAACGGCAGGT | Construction of plasmid pGADT7-opi1 |
| R-pGADT7-opi1 | GTTTTTCAGTATCTAAACCTTCTCCATTCCTGTCATGGAG | Construction of plasmid pGADT7-opi1 |
| F-pGBKT7-An01g13950 | CTGCATATGCCATCTGCACGACCTCCTGCCT | Construction of plasmid pGBKT7-An01g13950 |
| R-pGBKT7-An01g13950 | TGCAGGTCGACTTAGTTGCGAAACGAATCATCAATGCG | Construction of plasmid pGBKT7-An01g13950 |
| F-pGBKT7-An02g04350 | CATATGGAACTAAACCGGTCGGGACAACAT | Construction of plasmid pGBKT7-An02g04350 |
| R-pGBKT7-An02g04350 | GTCGACTTACCGGGGCATCACCAAGCCATTC | Construction of plasmid pGBKT7-An02g04350 |

**Table S5. Projects of yeast two hybrid experiments.**

| **Plasmids transformed Y187** | **Plasmids transformed Y2HGold** | **Description** |
| --- | --- | --- |
| pGADT7-An15g02370 | pGBKT7-An01g13950 | To verify the combination of *An01g13950* & *opi1* *in vitro* and *An02g04350* & *opi1* |
| pGADT7-An15g02370 | pGBKT7-An02g04350 |  |
| pGADT7-An03g04180 | pGBKT7-An02g04350 | To confirm the other bHLH protein that can form heterodimer with *ino2* |
| pGADT7-An03g05170 | pGBKT7-An02g04350 |  |
| pGADT7-An08g01380 | pGBKT7-An02g04350 |  |
| pGADT7-An08g04000 | pGBKT7-An02g04350 |  |
| pGADT7-An09g06630 | pGBKT7-An02g04350 |  |
| pGADT7-An14g02540 | pGBKT7-An02g04350 |  |
| pGADT7-An15g03940 | pGBKT7-An02g04350 |  |
| pGADT7-An01g13950 | pGBKT7-An02g04350 |  |

**Table S6 Quantitation results of peak area of WT and Δino2**

| **Sample** | **Quantitation of peak area** |
| --- | --- |
| WT-1 | 25500 |
| WT-2 | 31200 |
| WT-3 | 33500 |
| WT average | 30066 |
| Δino2-1 | 22400 |
| Δino2-2 | 21700 |
| Δino2-3 | 22700 |
| Δino2 average | 22266 |

**Table S7. *A. niger* Samples in RNA-seq.**

| **Samples** | **Strains** | **Descriptions** |
| --- | --- | --- |
| WT | WT | Spores of WT strain inoculated into liquid CD medium for 48h and extracted total RNA |
| Δino2 | Δino2 | Spores of Δino2 strain inoculated into liquid CD medium for 48h and extracted total RNA |
| INO | WT | Spores of WT strain inoculated into liquid CD + 100mg/mL inositol for 48h and extracted total RNA |

**Table S8. MALDI-TOF mass spectrometry results of pET22b-An01g13950 and pET22b-An02g04350 expressed in E. coli BL21(DE3)**

| **Sample** | **Accession** | **Description** | **Mass** | **Score** | **Matches** | **Sequencee** | **emPAI** | **Coverage** |
| --- | --- | --- | --- | --- | --- | --- | --- | --- |
| pET22b-An01g13950 | tr\|A2QB55\|A2QB55_ASPNC | Aspergillus niger contig An01c0450, genomic contig OS=Aspergillus niger (strain CBS 513.88 / FGSC A1513) GN=An01g13950 PE=4 SV=1 | 15879 | 7634 | 754(423) | 10(8) | 12.89 | 57% |
| pET22b-An02g04350 | tr\|A0A254TXT9\|A0A254TXT9_ASPNG | Helix-loop-helix DNA-binding domain family  protein OS=Aspergillus niger GN=CAN33_54150 PE=4 SV=1 | 31785 | 712 | 72(24) | 8(5) | 1.46 | 46% |

**Table S9 Differentially expressed UASino genes in A. niger**

| Gene name in *S. cerevisiae* | Description | Sequence Hits in *A. niger* | Score (bits) | E-value | Normalized expression level | | | | | | |
| --- | --- | --- | --- | --- | --- | --- | --- | --- | --- | --- | --- |
|  |  |  |  |  | **WT** | **Δino2** | **INO** | **Δino2/WT** | | **INO/WT** | |
|  |  |  |  |  |  |  |  | **Log_2_FC** | **Q-value** | **Log_2_FC** | **Q-value** |
| cds1 | Phosphatide cytidylyltransferase (CDP-diglyceride synthetase) | An07g09570 | 399 | 8.00E-112 | 49.095 | 37.595 | 37.25 | -0.3810367 | 2.69014E-12 | -0.395975179 | 3.35108E-14 |
| cho1 | Phosphatidylserine synthase | An01g09480 | 200 | 4.00E-52 | 107.64 | 168.395 | 109.045 | 0.642600769 | 2.62336E-49 | 0.019367791 | 0.310170624 |
| cho2 | Phosphatidylethanolamine methyltransferase (PEMT) | An15g06310 | 429 | 2.00E-120 | 153.83 | 180.225 | 139.84 | 0.22837352 | 5.08273E-37 | -0.136239385 | 6.04669E-14 |
| cki1 | Choline kinase | An01g11150 | 235 | 2.00E-62 | 30.89 | 29.785 | 22.6 | -0.055452496 | 0.082183531 | -0.448294525 | 1.22749E-17 |
| cpt1-1 | Choline phosphotransferase | An11g09660 | 239 | 9.00E-64 | 30.02 | 23.56 | 20.035 | -0.351029227 | 1.01421E-06 | -0.578731167 | 1.73829E-15 |
| cpt1-2 | Choline phosphotransferase | An16g07870 | 203 | 1.00E-52 | 20.53 | 15.62 | 11.44 | -0.388607582 | 1.2298E-05 | -0.841084282 | 5.62568E-19 |
| eki1-1 | Ethanolamine kinase | An01g11150 | 157 | 9.00E-39 | 30.89 | 29.785 | 22.6 | -0.055452496 | 0.082183531 | -0.448294525 | 1.22749E-17 |
| eki1-2 | Ethanolamine kinase | An08g03670 | 82 | 3.00E-16 | 47.955 | 35.355 | 34.475 | -0.441771185 | 2.38879E-17 | -0.474143939 | 1.84002E-21 |
| ept1-1 | sn-1,2-diacylglycerol ethanolamine- and choline phosphotransferase | An11g09660 | 254 | 3.00E-68 | 30.02 | 23.56 | 20.035 | -0.351029227 | 1.01421E-06 | -0.578731167 | 1.73829E-15 |
| ept1-2 | sn-1,2-diacylglycerol ethanolamine- and choline phosphotransferase | An16g07870 | 191 | 4.00E-49 | 20.53 | 15.62 | 11.44 | -0.388607582 | 1.2298E-05 | -0.841084282 | 5.62568E-19 |
| hnm1-1 | Plasma membrane transporter for choline, ethanolamine, and carnitine | An09g05010 | 528 | 2.00E-150 | 77.295 | 45.285 | 75.08 | -0.775451806 | 2.81081E-70 | -0.038689595 | 0.158326881 |
| hnm1-2 | Plasma membrane transporter for choline, ethanolamine, and carnitine | An15g01900 | 513 | 6.00E-146 | 7.28 | 5.38 | 9.965 | -0.442592244 | 0.000437574 | 0.461332241 | 2.22403E-05 |
| hnm1-3 | Plasma membrane transporter for choline, ethanolamine, and carnitine | An16g07900 | 299 | 1.00E-81 | 4.99 | 7.275 | 6.575 | 0.543840144 | 2.36084E-05 | 0.401516996 | 0.001670317 |
| ino1 | Inositol-3-phosphate synthase | An10g00530 | 590 | 4.00E-169 | 975.305 | 748.325 | 400.65 | -0.381464777 | 5.0519E-249 | -1.283032639 | 0 |
| itr1-1 | Myo-inositol transporter | An04g00340 | 386 | 1.00E-107 | 17.285 | 14.665 | 13.16 | -0.234577647 | 0.001713335 | -0.390982321 | 9.72424E-07 |
| itr1-2 | Myo-inositol transporter | An12g09370 | 380 | 8.00E-106 | 3.915 | 4.25 | 3.88 | 0.105813069 | 0.135079487 | -0.014096314 | 0.392772893 |
| itr1-3 | Myo-inositol transporter | An05g00490 | 357 | 5.00E-99 | 20.38 | 11.37 | 23.84 | -0.839938345 | 3.40643E-40 | 0.230826748 | 3.45049E-06 |
| opi3 | Methylene-fatty-acyl-phospholipid synthase | An08g00560 | 229 | 5.00E-61 | 181.22 | 114.72 | 128.045 | -0.653767812 | 7.12672E-62 | -0.499130239 | 3.37701E-43 |
| psd1 | Phosphatidylserine decarboxylase of the mitochondrial inner membrane | An01g14110 | 320 | 7.00E-88 | 155.4 | 104.365 | 166.24 | -0.570192224 | 3.73889E-86 | 0.099418684 | 4.4051E-05 |
| pct1 | Cholinephosphate cytidylyltransferase | An08g00840 | 312 | 2.0e-85 | 153.545 | 156.73 | 135.11 | 0.028768 | 0.092673816 | -0.18313 | 2.06E-10 |
| dga1 | Diacylglycerol acyltransferase | An13g00040 | 272 | 1.0e-73 | 37.82 | 22.39 | 33.105 | -0.7515 | 1.26035E-26 | -0.18974 | 0.00143 |
| lro1 | Acyltransferase that catalyzes diacylglycerol esterification | An16g03640 | 517 | 5.0e-147 | 27.395 | 25.76 | 25.795 | -0.09436 | 0.035616576 | -0.08529 | 0.07882 |
| are1/are2 | Acyl-CoA:sterol acyltransferase | An18g04660 | 194 | 5.0e-50 | 15.945 | 11.655 | 13.01 | -0.44796 | 1.40787E-07 | -0.2925 | 0.000233 |
| ino2 | Transcription factor | An04g04350 | - | - | 17.965 | 0 | 15.895 | -9.904758012 | 1.47218E-96 | -0.175725918 | 0.024590541 |
| acc1 | Acetyl-CoA carboxylase | An12g04020 | 2914 | 0.0e+00 | 119.105 | 216.395 | 104.26 | 0.863408 | 0 | -0.19213 | 1.17E-41 |
|  |  |  |  |  |  |  |  | 16/22  Repressed |  | 17/22 Repressed |  |

**Table S10 Differentially expressed fatty acid synthase genes in A. niger**

| Gene name in *S. cerevisiae* | Description | Sequence Hits in *A. niger* | Score (bits) | E-value | Normalized expression level | | | | | | |
| --- | --- | --- | --- | --- | --- | --- | --- | --- | --- | --- | --- |
|  |  |  |  |  | **WT** | **Δino2** | **INO** | **Δino2/WT** | | **INO/WT** | |
|  |  |  |  |  |  |  |  | **Log_2_FC** | **Q-value** | **Log_2_FC** | **Q-value** |
| fas1-1 | Beta subunit of fatty acid synthetase | An08g10860 | 1451 | 0.00E+00 | 15.775 | 940.025 | 10.63 | 5.901479124 | 0 | -0.5707655 | 1.3525E-37 |
| fas1-2 | Beta subunit of fatty acid synthetase | An09g01740 | 1417 | 0.00E+00 | 0.74 | 1.435 | 1.18 | 0.938149415 | 8.86986E-08 | 0.67097767 | 0.00014178 |
| fas1-3 | Beta subunit of fatty acid synthetase | An10g00650 | 1391 | 0.00E+00 | 0.135 | 2.61 | 0.155 | 4.279846181 | 2.08766E-88 | 0.20149239 | 0.29255224 |
| fas1-4 | Beta subunit of fatty acid synthetase | An12g01990 | 1314 | 0.00E+00 | 2.535 | 2.035 | 3.455 | -0.322975401 | 0.001504093 | 0.44676251 | 1.2695E-06 |
| fas1-5 | Beta subunit of fatty acid synthetase | An01g00050 | 1286 | 0.00E+00 | 133.035 | 139.325 | 64.03 | 0.074706125 | 3.04548E-05 | -1.0518279 | 0 |
| fas1-6 | Beta subunit of fatty acid synthetase | An09g01750 | 333 | 4.00E-91 | 2.53 | 6.82 | 5.94 | 1.413990193 | 1.97277E-11 | 1.23144561 | 3.3048E-09 |
| fas2-1 | Alpha subunit of fatty acid synthetase | An01g00060 | 2385 | 0.00E+00 | 214.68 | 291.85 | 186.82 | 0.443580272 | 0 | -0.1987058 | 1.8858E-64 |
| fas2-2 | Alpha subunit of fatty acid synthetase | An08g10930 | 1298 | 0.00E+00 | 13.605 | 1051.515 | 7.625 | 6.274185942 | 0 | -0.8391465 | 2.2142E-56 |
| fas2-3 | Alpha subunit of fatty acid synthetase | An09g02010 | 1288 | 0.00E+00 | 0.08 | 0.7 | 0.185 | 3.087761961 | 1.7722E-15 | 1.21154605 | 0.01313324 |
| fas2-4 | Alpha subunit of fatty acid synthetase | An12g01980 | 1197 | 0.00E+00 | 0.88 | 0.725 | 1.135 | -0.288775827 | 0.047290625 | 0.37058274 | 0.02086361 |
| fas2-5 | Alpha subunit of fatty acid synthetase | An10g00630 | 1181 | 0.00E+00 | 0.18 | 3.24 | 0.115 | 4.213554502 | 7.70439E-84 | -0.5920567 | 0.12221393 |
| acc1 | Acetyl-CoA carboxylase | An12g04020 | 2914 | 0.00E+00 | 119.105 | 216.395 | 104.26 | 0.863408 | 0 | -0.19213 | 1.17E-41 |
|  |  |  |  |  |  |  |  | 10/12 Induced |  | 6/12 Induced |  |

| Gene name in *S. cerevisiae* | Description | Sequence Hits in *A. niger* | Score (bits) | E-value | Normalized expression level | | | | | | |
| --- | --- | --- | --- | --- | --- | --- | --- | --- | --- | --- | --- |
|  |  |  |  |  | **WT** | **Δino2** | **INO** | **Δino2/WT** | | **INO/WT** | |
|  |  |  |  |  |  |  |  | **Log_2_FC** | **Q-value** | **Log_2_FC** | **Q-value** |
| ino1 | Inositol-3-phosphate synthase | An10g00530 | 590 | 4.00E-169 | 975.305 | 748.325 | 400.65 | -0.38146 | 5.1E-249 | -1.28303 | 0 |
| inm1 | Inositol monophosphates | An03g03700 | 173 | 5.00E-44 | 89.845 | 146.19 | 139.435 | 0.69846122 | 3.8497E-70 | 0.63495296 | 7.14193E-64 |
| pis1 | Phosphatidylinositol synthase | An01g14140 | 179 | 5.00E-46 | 87.41 | 92.24 | 134.915 | 0.08011466 | 0.03137853 | 0.6279945 | 4.59424E-52 |

**Table S11 Differentially expressed PI synthesis pathway genes in A. niger**

**Table S12 Differentially expressed PI metabolism genes in A. niger**

| Gene identifier | Gene name in *S. cerevisiae* | Description | Normalized expression level | | | | | | |
| --- | --- | --- | --- | --- | --- | --- | --- | --- | --- |
|  |  |  | **Δino2** | **WT** | **INO** | **Δino2/WT** | | **INO/WT** | |
|  |  |  |  |  |  | **Log_2_FC** | **Q-value** | **Log_2_FC** | **Q-value** |
| GO: 0006661 phosphatidylinositol biosynthetic process | | |  |  |  |  |  |  |  |
| An04g07010  An16g07540 | caM  vps30 | Calmodulin  Subunit of phosphatidylinositol (PtdIns) 3-kinase complexes I and II | 671.9  10.325 | 507.265  11.135 | 446.1  12.63 | 0.403142  -0.11621 | 1.62793E-51  0.070032638 | -0.18441  0.183728 | 4.75E-11  0.025966 |
| An08g10570 | vac14 | Enzyme regulator; involved in synthesis of phosphatidylinositol 3,5-bisphosphate | 30.105 | 37.87 | 32.975 | -0.33012 | 2.30128E-14 | -0.19713 | 9.62E-07 |
| An07g01640 | cmd1 | Calmodulin; Ca2+ binding protein that regulates Ca2+ independent processes | 195.4 | 217.07 | 201.825 | -0.15297 | 0.000375819 | -0.10367 | 0.012086 |
| GO: 0010513 positive regulation of phosphatidylinositol biosynthetic process | | |  |  |  |  |  |  |  |
| An02g07780 | arf3 | Glucose-repressible ADP-ribosylation factor | 64.62 | 104.815 | 78.055 | -0.6928 | 8.35053E-28 | -0.42282 | 1.77E-13 |
| GO: 0010512 negative regulation of phosphatidylinositol biosynthetic process | | |  |  |  |  |  |  |  |
| An01g09480 | cho1 | Phosphatidylserine synthase | 168.395 | 107.64 | 109.045 | 0.642601 | 2.62336E-49 | 0.019368 | 0.310171 |

**Table S13 Differentially expressed ERAD genes in A. niger**

| Gene name in *S. cerevisiae* | Description | Sequence Hits in *A. niger* | Score (bits) | E-value | Normalized expression level | | | | | | |
| --- | --- | --- | --- | --- | --- | --- | --- | --- | --- | --- | --- |
|  |  |  |  |  | **WT** | **Δino2** | **INO** | **Δino2/WT** | | **INO/WT** | |
|  |  |  |  |  |  |  |  | **Log_2_FC** | **Q-value** | **Log_2_FC** | **Q-value** |
| cwh41 | Processing alpha glucosidase I | An15g01420 | 514 | 4.00E-146 | 33.64 | 20.245 | 24.32 | -0.73302 | 5.07E-45 | -0.46636 | 3.86E-23 |
| der1 | ER membrane protein that promotes export of misfolded polypeptides requires N-terminal acetylation by NatB | An15g00640 | 60 | 4.00E-10 | 20.675 | 16.58 | 20.335 | -0.32015 | 0.002113 | -0.02278 | 0.360918 |
| gtb1 | Glucosidase II beta subunit, forms a complex with alpha subunit Rot2p | An13g00620 | 85 | 4.00E-17 | 93.96 | 65.545 | 83.305 | -0.51987 | 1.04E-46 | -0.17204 | 1.01E-07 |
| hrd1 | Ubiquitin-protein ligase involved in ER-associated degradation (ERAD) of misfolded proteins | An16g07970 | 129 | 2.00E-30 | 52.675 | 32.865 | 47.055 | -0.67488 | 2E-52 | -0.16131 | 2.46E-05 |
| hrd3 | ER membrane protein that plays a central role in ERAD | An01g12720 | 160 | 1.00E-39 | 76.455 | 43.75 | 65.245 | -0.8035 | 4.39E-98 | -0.22556 | 6.15E-12 |
| mnl1-1 | Alpha-1,2-specific exo-mannosidase of the endoplasmic reticulum | An12g00340 | 333 | 2.00E-91 | 14.18 | 8.625 | 10.78 | -0.71602 | 2.21E-22 | -0.39441 | 2.38E-09 |
| mnl1-2 | Alpha-1,2-specific exo-mannosidase of the endoplasmic reticulum | An18g06220 | 124 | 1.00E-28 | 30.93 | 21.32 | 20.925 | -0.54037 | 1.16E-18 | -0.55999 | 9E-22 |
| mnl1-3 | Alpha-1,2-specific exo-mannosidase of the endoplasmic reticulum | An04g06990 | 111 | 9.00E-25 | 46.66 | 33.715 | 39.925 | -0.45563 | 1.16E-19 | -0.2211 | 1.35E-06 |
| rot2 | Glucosidase II catalytic subunit | An09g05880 | 677 | 0.00E+00 | 51.04 | 31.185 | 32.605 | -0.71024 | 3.49E-74 | -0.64487 | 2.03E-70 |
| ssm4 | Membrane-embedded ubiquitin-protein ligase | An12g04000 | 125 | 1.00E-28 | 33.65 | 23.31 | 28.345 | -0.53037 | 1.05E-46 | -0.24629 | 1.55E-13 |
| ubc7-1 | Ubiquitin conjugating enzyme | An09g06110 | 213 | 2.00E-56 | 98.685 | 142.87 | 117.155 | 0.535552 | 7.21E-22 | 0.249514 | 6.81E-06 |
| ubc7-2 | Ubiquitin conjugating enzyme | An06g01040 | 158 | 7.00E-40 | 138.67 | 288.045 | 130.62 | 1.054328 | 2.3E-134 | -0.08596 | 0.052863 |
| ubc7-3 | Ubiquitin conjugating enzyme | An06g01120 | 130 | 2.00E-31 | 176.56 | 186.29 | 191.705 | 0.074511 | 0.037469 | 0.120617 | 0.005417 |
| ubx2 | Bridging factor involved in ER-associated protein degradation (ERAD) | An01g12370 | 73 | 2.00E-13 | 34.14 | 37.67 | 41.72 | 0.141677 | 0.004257 | 0.289371 | 1.95E-08 |
| ufd2 | Ubiquitin chain assembly factor (E4) | An04g01730 | 481 | 3.00E-136 | 66.01 | 72.73 | 56.255 | 0.138647 | 3.67E-07 | -0.22849 | 5.4E-16 |
| sel1 | Bridging factor involved in ER-associated protein degradation (ERAD) | An01g12370 | 73 | 2.00E-13 | 34.14 | 37.67 | 41.72 | 0.141677 | 0.004257 | 0.289371 | 1.95E-08 |
|  |  |  |  |  |  |  |  | 10/16 Repressed |  | 12/16 Repressed |  |

| Gene name in *S. cerevisiae* | Description | Sequence Hits in *A. niger* | Score (bits) | E-value | Normalized expression level | | | | | | |
| --- | --- | --- | --- | --- | --- | --- | --- | --- | --- | --- | --- |
|  |  |  |  |  | **WT** | **Δino2** | **INO** | **Δino2/WT** | | **INO/WT** | |
|  |  |  |  |  |  |  |  | **Log_2_FC** | **Q-value** | **Log_2_FC** | **Q-value** |
| chs3 | Chitin synthase III | An09g02290 | 1154 | 0.00E+00 | 51.44 | 42.12 | 41.715 | -0.28556 | 4.86E-19 | -0.30145 | 4.92E-23 |
| chs5 | Component of the exomer complex; the exomer which also contains Csh6p, Bch1p, Bch2p, and Bud7 | An12g07880 | 251 | 4.00E-67 | 51.99 | 40.5 | 43.575 | -0.36113 | 4.63E-11 | -0.25305 | 8.86E-07 |
| chs7 | May be involved in chitin biosynthesis by regulation of Chs3p export from the ER | An08g04350 | 271 | 2.00E-73 | 37.315 | 26.51 | 31.235 | -0.48935 | 5.69E-11 | -0.25379 | 0.000193 |
| skt5-1 | Activator of Chs3p (chitin synthase III) | An12g05030 | 283 | 1.00E-76 | 78.185 | 73.83 | 70.975 | -0.08287 | 0.00473 | -0.13859 | 1.16E-05 |
| skt5-2 | Activator of Chs3p (chitin synthase III) | An12g07540 | 215 | 4.00E-56 | 31.175 | 24.85 | 28.935 | -0.32964 | 5.67E-12 | -0.10707 | 0.011127 |
| skt5-3 | Activator of Chs3p (chitin synthase III) | An11g11050 | 126 | 2.00E-29 | 104.065 | 93.895 | 75.97 | -0.14592 | 1.12E-07 | -0.4527 | 4.94E-59 |
|  |  |  |  |  |  |  |  | 6/6 Repressed |  | 6/6 Repressed |  |

**Table S14 Differentially expressed chitin synthesis genes in A. niger**

**Table S15 Differentially expressed DNA damage response genes in A. niger**

| Gene name in *S. cerevisiae(S) or in A. nidulans(A)* | Description | Sequence Hits in *A. niger* | Score (bits) | E-value | Normalized expression level | | | | | | |
| --- | --- | --- | --- | --- | --- | --- | --- | --- | --- | --- | --- |
|  |  |  |  |  | **WT** | **Δino2** | **INO** | **Δino2/WT** | | **INO/WT** | |
|  |  |  |  |  |  |  |  | **Log_2_FC** | **Q-value** | **Log_2_FC** | **Q-value** |
| top1(S) | Putative topoisomerase I | An01g04640 | 785 | 0.00E+00 | 97.53 | 82.72 | 67.67 | -0.23831 | 9.61E-19 | -0.52508 | 1.56E-85 |
| cshA(A) | Protein with homology to Cockayne's syndrome nucleotide excision repair protein; transcript upregulated in response to camptothecin | An14g01060 | 1824 | 0.00E+00 | 7.29 | 6.41 | 10.02 | -0.18892 | 0.008518 | 0.461124 | 6.34E-11 |
| fhdA(A) | Putative forkhead-associated domain protein; transcript upregulated in response to camptothecin | An14g03210 | 323 | 6.00E-89 | 13.185 | 11.25 | 12.3 | -0.22433 | 0.021344 | -0.09642 | 0.202385 |
| mshA(A) | Putative MutS homolog with a predicted role in DNA mismatch repair; transcript upregulated in response to camptothecin | An04g03050 | 2031 | 0.00E+00 | 14.04 | 12.65 | 8.925 | -0.14953 | 0.005002 | -0.653 | 1.04E-25 |
| tprA-1(A) | Tetratricopeptide repeat (TPR)-containing protein; usvB-dependent transcript induction in response to camptothecin | An03g03030 | 555 | 3.00E-158 | 26.24 | 17.85 | 22.29 | -0.55448 | 3.72E-37 | -0.23091 | 3.26E-09 |
| tprA-2(A) | Tetratricopeptide repeat (TPR)-containing protein; usvB-dependent transcript induction in response to camptothecin | An08g11610 | 401 | 7.00E-112 | 12.88 | 11.36 | 12.36 | -0.17909 | 0.002975 | -0.05845 | 0.185015 |
| tprA-3(A) | Tetratricopeptide repeat (TPR)-containing protein; usvB-dependent transcript induction in response to camptothecin | An19g00300 | 300 | 2.00E-81 | 52.95 | 49.24 | 50.755 | -0.09919 | 0.01666 | -0.05921 | 0.121707 |
| uvsC-1(A) | Involved in the DNA damage response; mutants are sensitive to DNA double-strand breaks and have defects in homologous recombination | An08g02350 | 694 | 0.00E+00 | ND | ND | ND | - | - | - | - |
| uvsC-2(A) | Involved in the DNA damage response; mutants are sensitive to DNA double-strand breaks and have defects in homologous recombination | An12g00460 | 281 | 2.00E-76 | 3.42 | 3.035 | 4.115 | -0.16659 | 0.12663 | 0.272893 | 0.10928 |
|  |  |  |  |  |  |  |  | 7/7 Repressed |  | 5/7 Repressed |  |

1. **Supplementary figures**

**
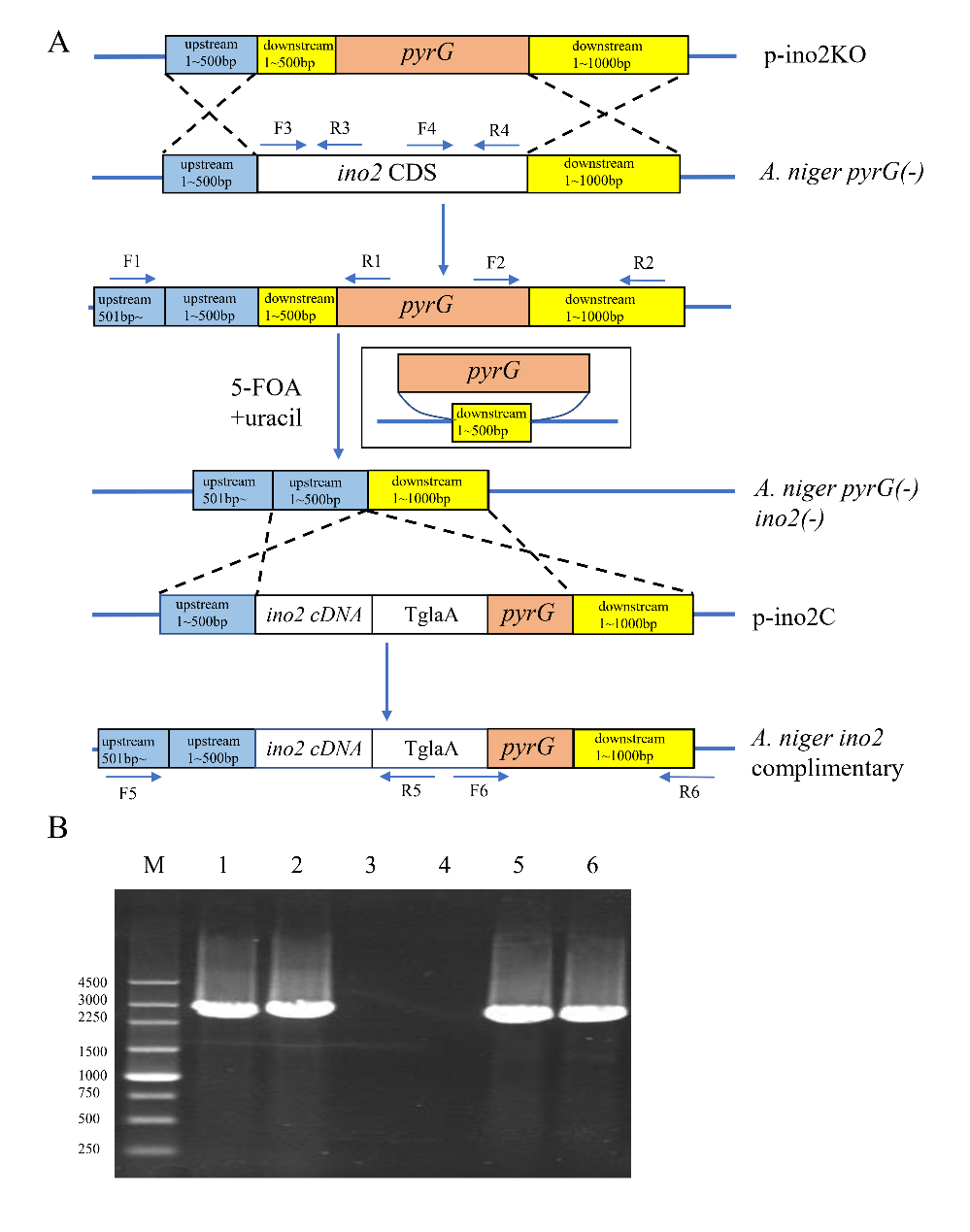
**

**Figure S1. Schematic of disruption and complementation of ino2 in A. niger.**

(A) Homologous direct repair was used to disrupt the *ino2* CDS. The marker of *pyrG* was then recycled and used for the complementation of *ino2* cDNA. The blue rectangle represented the upstream of *ino2* CDS (before the start codon) and the yellow rectangle represented the downstream of *ino2* CDS (after the stop codon). Downstream 1~500bp of *ino2* CDS was used to recycle the marker of *pyrG*. (B) PCR amplification for verification of the disruption and complementation of *ino2* exhibited in (A). Line1(F1/R1) and line 2(F2/R2) were the bands for the location of upstream and downstream of *ino2* disruptant framework (p-ino2KO ). Line 3(F3/R3) and line 4 (F4/R4) were the results for the amplification of *ino2* CDS. Line5(F5/R5) and line6(F6/R6) were the bands for the verification of location of *ino2* complementation framework. M: marker. Correct bands of each sets of primers were: F1/R1, 2782 bp; F2/R2, 2819 bp; F3/R3, 565 bp; F4/R4, 557 bp; F5/R5, 2382 bp; F6/R6, 2394 bp.

**
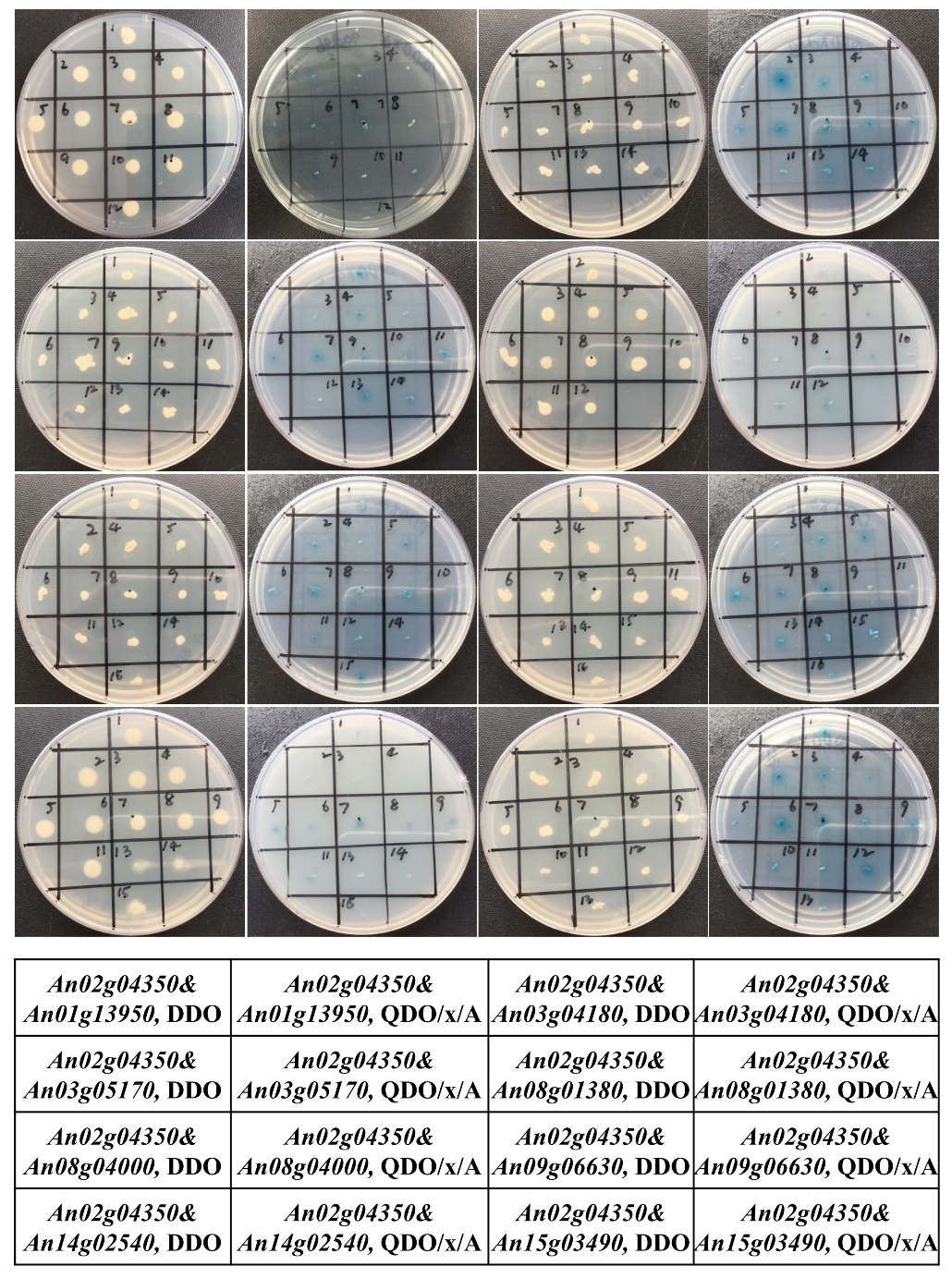
**

**Figure S2 Confirmation of interaction of An02g04350 and 8 bHLH transcription factors by yeast two hybrid assay.**

The plasmid pGBKT7-An02g04350 was transformed into the *S. cerevisiae* Y2HGold strain, and pGADT7-An01g13950, pGADT7-An03g04180, pGADT7-An03g05170, pGADT7-An08g01380, pGADT7-An08g04000, pGADT7-An09g06630, pGADT7-An14g02540, pGADT7-An15g03490, and pGADT7-opi1 were transformed into the *S. cerevisiae* Y187 strain. Transformed yeast cells were grown on DDO selective solid medium and further inoculated on QDO/x/A selected solid medium. Table at the bottom marked out the pGADT7 plasmids transformed into the Y187 strain and the types of selective solid medium.

**Figure S3 Results of intensity ration of LC-MS/MS of WT and Δino2.** The intensity ration was measured with 3 biological replicates with each sample.

**WT-1**

**
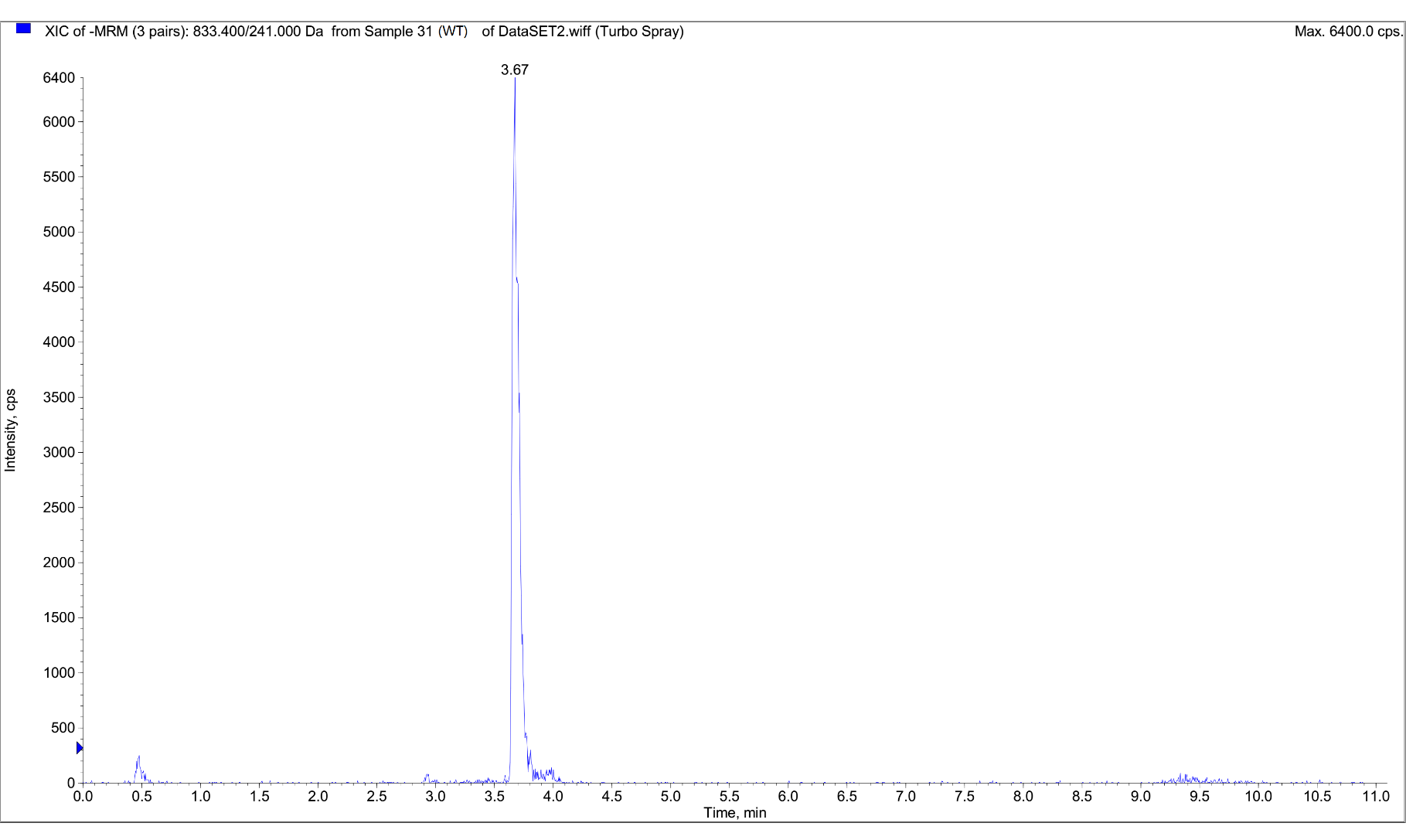
**

**WT-2**

**
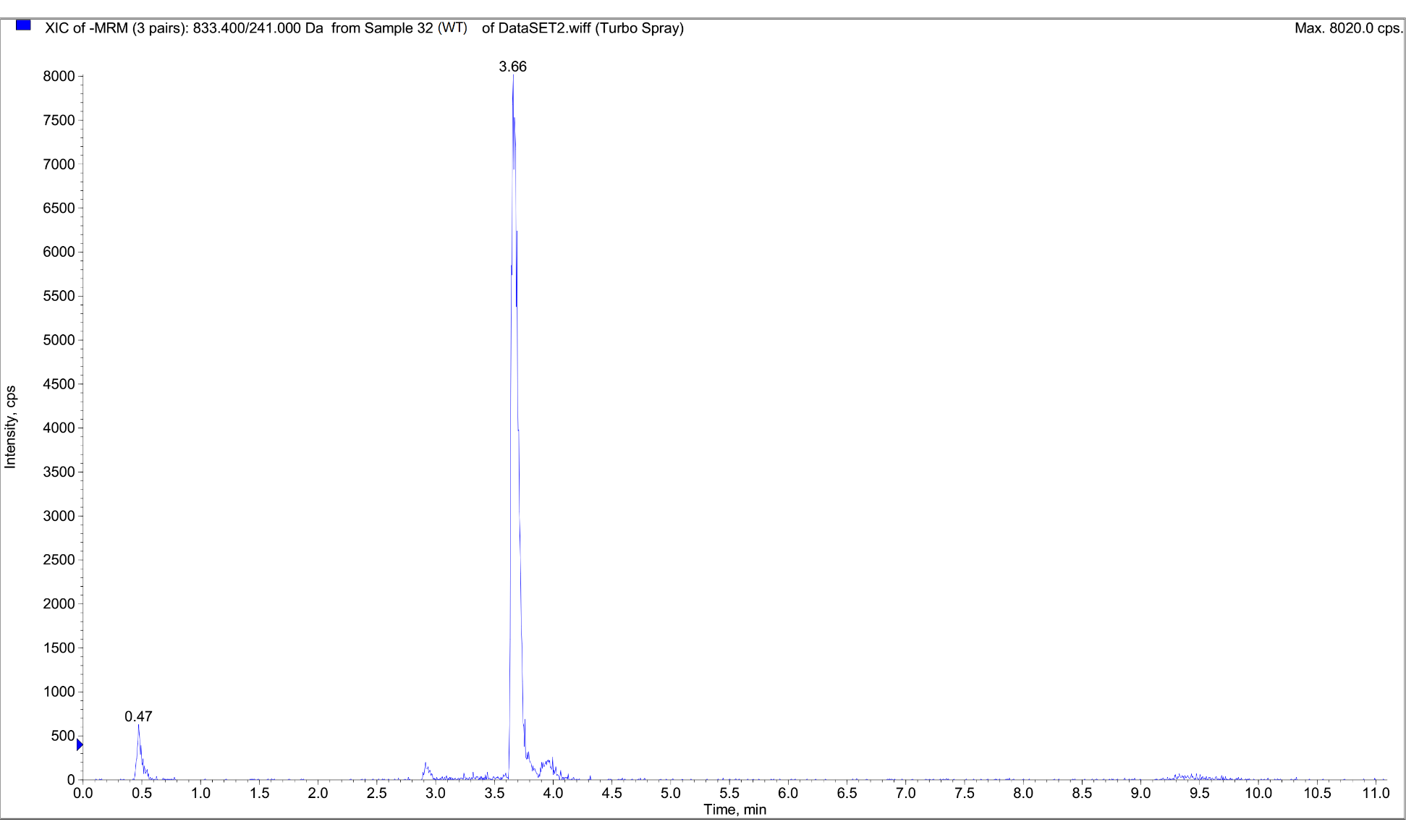
**

**WT-3**

**
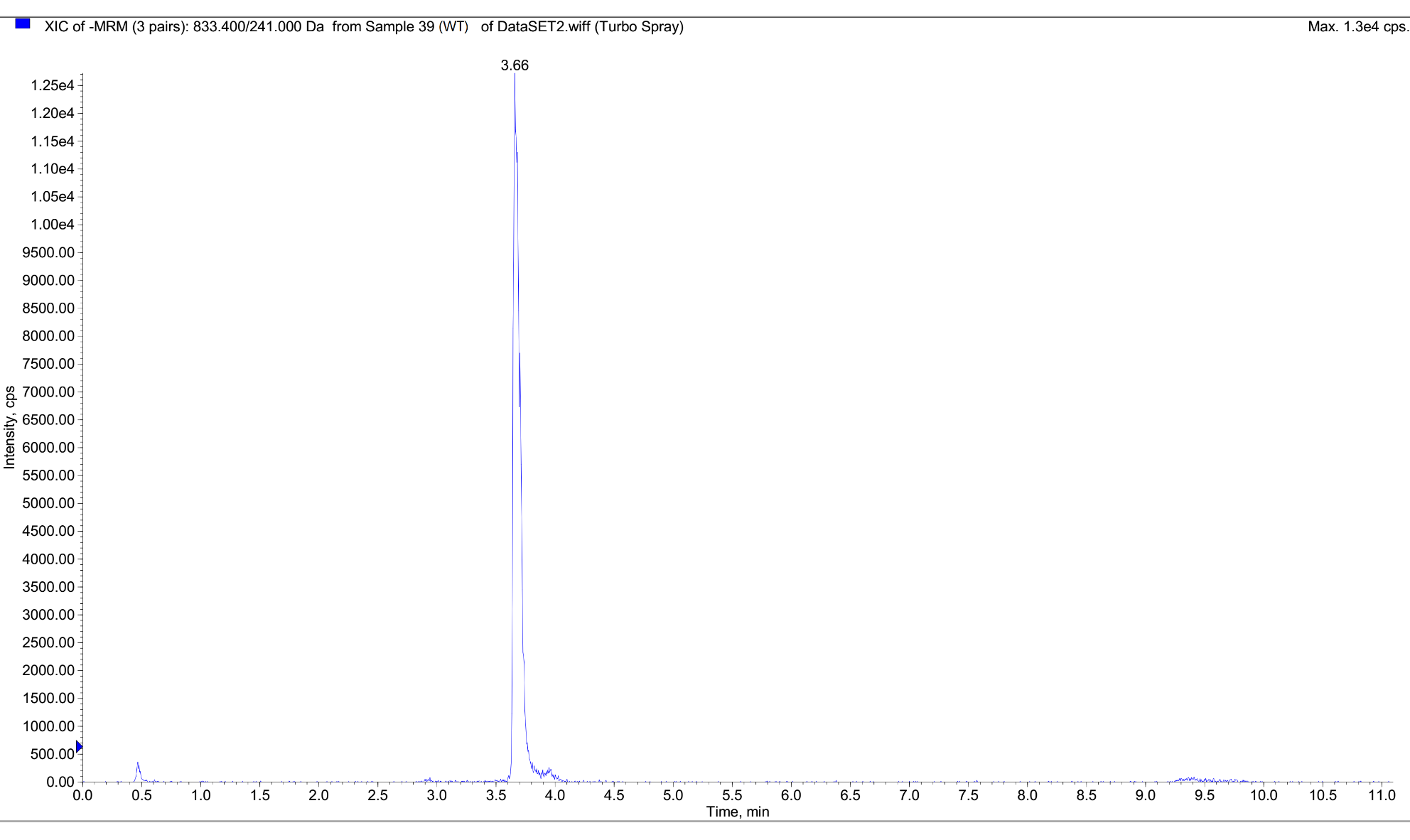
**

**Δino2-1**

**
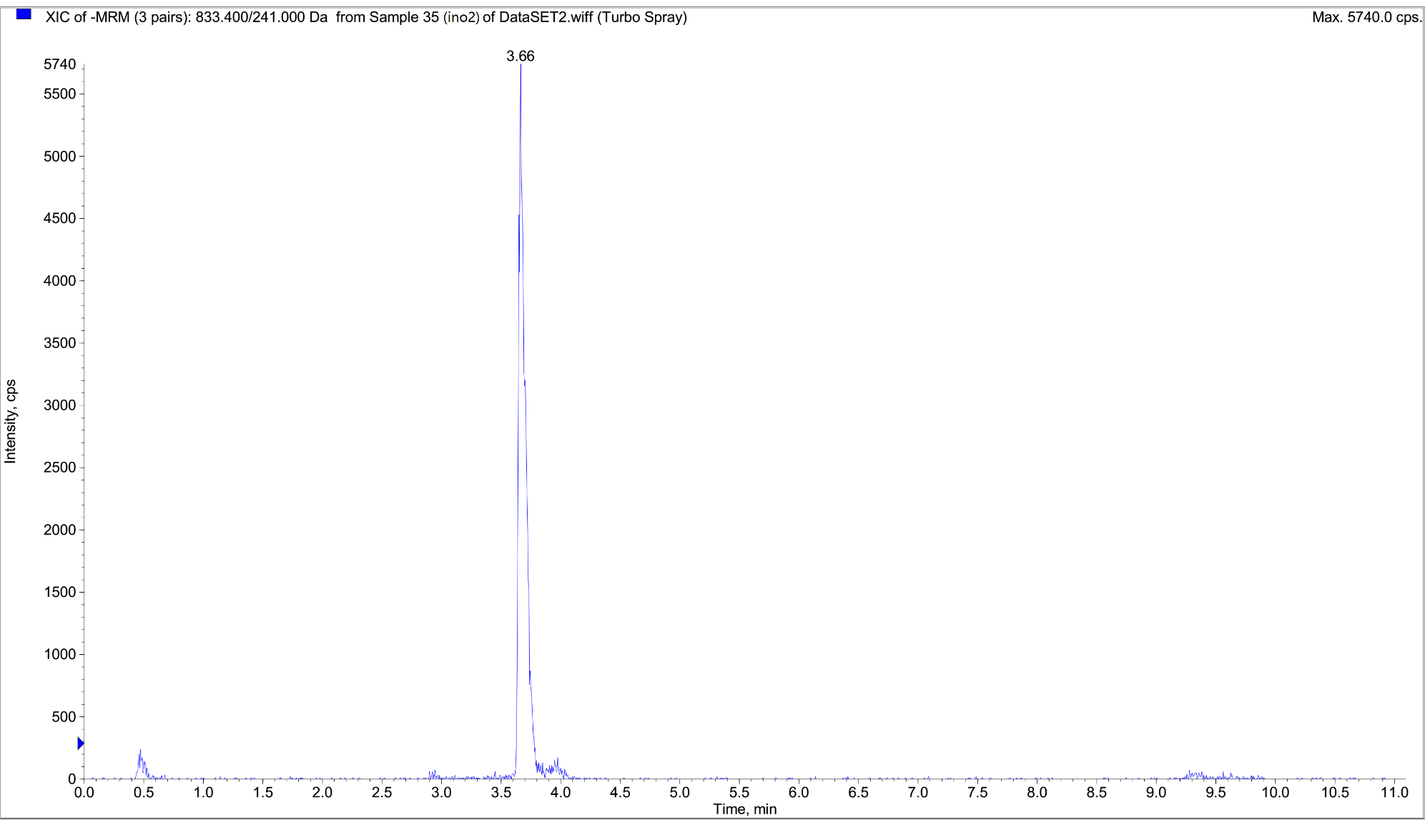
**

**Δino2-2**

**
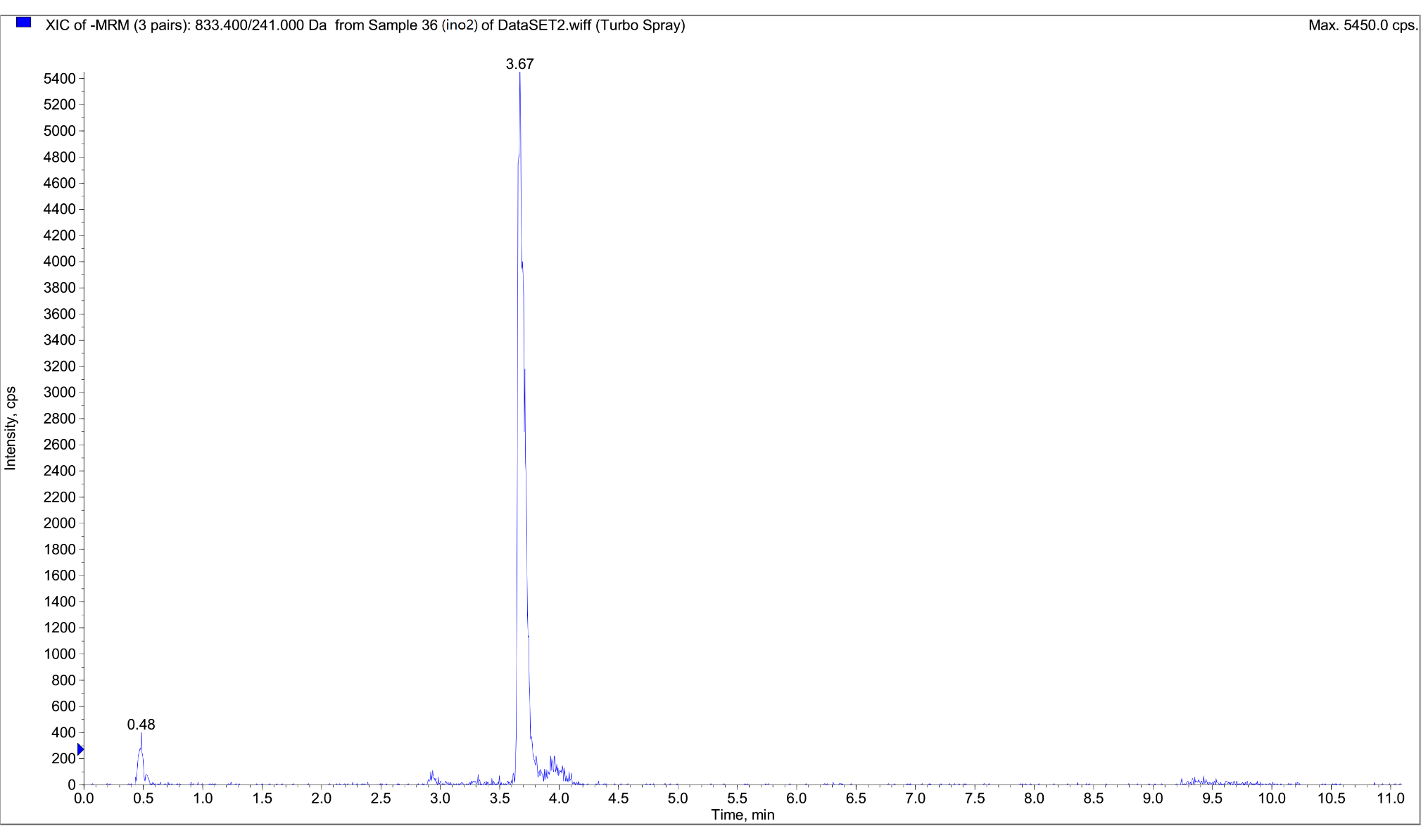
**

**Δino2-3**

**
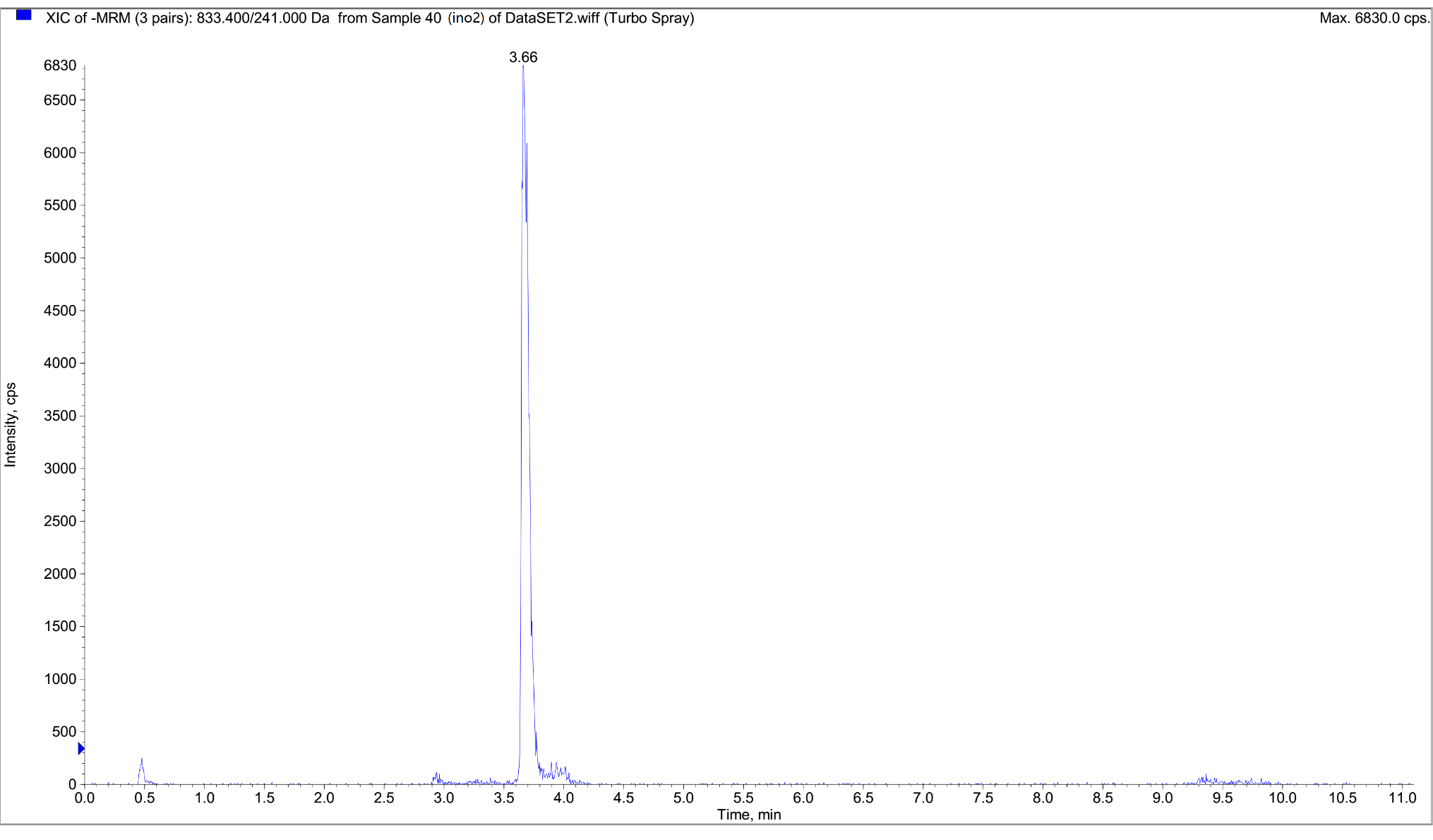
**

**
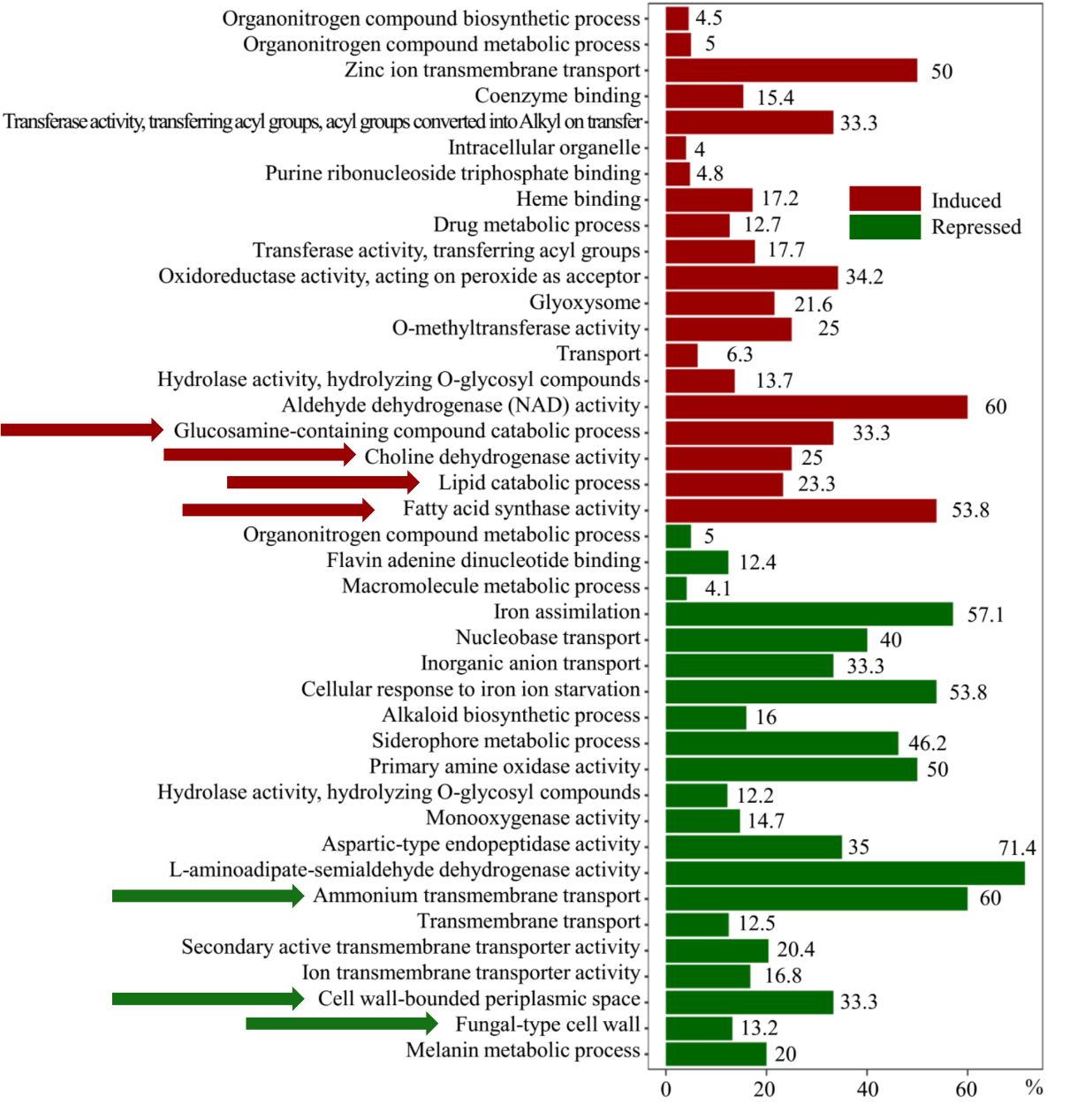
**

**Figure S4** GO function classification analysis of differentially induced or repressed genes in the Δino2 strain. Representation of the main significantly induced and repressed pathways (FDR＜0.05) in the Δino2 mutant strain compared with the WT strain.

**
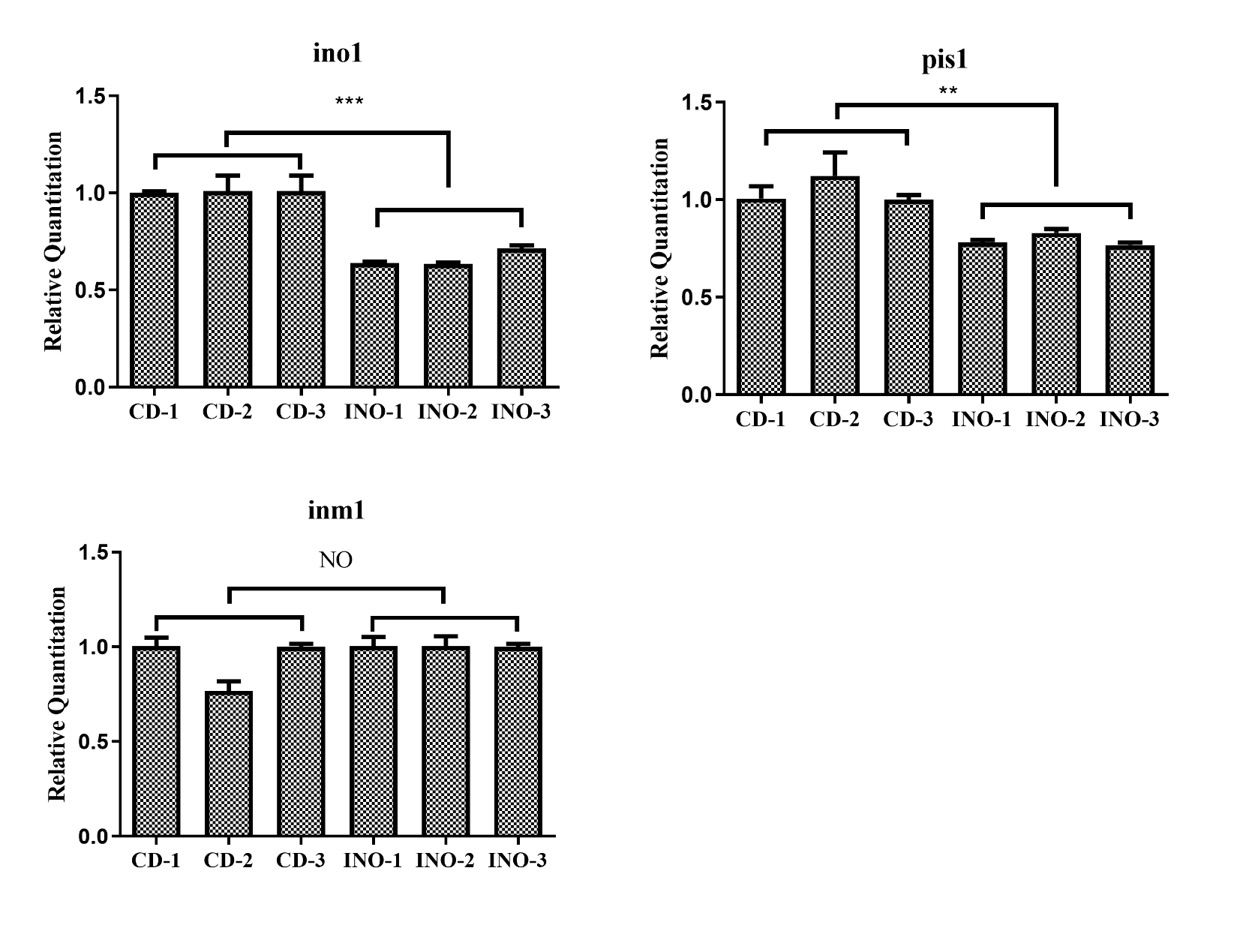
**

**Figure. S5** Comparative qRT-PCR of target genes (ino1, pis1 and inm1) between Δino2/CD and Δino2/INO. The relative expression levels were examined and compared using qRT-PCR. The expression levels were normalized to the expression level of the endogenous control gene *gpdA*. The expression value in Δino2/CD was used as the baseline. Each experiment was performed with 3 biological replicates and 4 technical replicates. ***, *p* <0.0005, **, *p* < 0.005, NO, non-significant. compared with the Δino2/CD strain. *p* values were determined using analysis of Student's *t* test.
